# Supplementary material for: Chemical Constituents of the Deep-Sea-Derived Penicillium citreonigrum MCCC 3A00169 and Their Antiproliferative Effects
Source: Mar Drugs. 2022 Nov 24;20(12):736. doi: 10.3390/md20120736 (PMC9781865; doi:10.3390/md20120736)
Supplement: Supplementary file 1 [file marinedrugs-20-00736-s001.zip › marinedrugs-2014757-supplementary.pdf]

Supporting Information

Chemical Constituents of the Deep-Sea-Derived *Penicillium citreonigrum* MCCC 3A00169 and Their Antiproliferative Effects

Zheng-Biao Zou [1,2,†], Gang Zhang [3,†], Yu-Qi Zhou [4,†], Chun-Lan Xie [2], Ming-Min Xie [2], Lin Xu [2], You-Jia Hao [2], Lian-Zhong Luo [3], Xiao-Kun Zhang [4,*], Xian-Wen Yang [2,*], and Jun-Song Wang [1,*]

[1] Center for Molecular Metabolism, School of Environmental and Biological Engineering, Nanjing University of Science and Technology, 200 Xiaolingwei Street, Nanjing 210094, China

[2] Key Laboratory of Marine Genetic Resources, Third Institute of Oceanography, Ministry of Natural Resources, 184 Daxue Road, Xiamen 361005, China

[3] Xiamen Key Laboratory of Marine Medicinal Natural Products Resources; Fujian Province Universities and Colleges Engineering Research Center for Marine Biomedical Resource Utilization; Xiamen Medica College, 1999 Guankouzhong Road, Xiamen 361023, China

[4] School of Pharmaceutical Sciences, Xiamen University, South Xiangan Road, Xiamen, 361102, China

* Correspondence: wang.junsong@gmail.com (J.-S.W.); xkzhang@xmu.edu.cn (X.-K.Z.); yangxianwen@tio.org.cn (X.-W.Y.); Tel.: +86-25- 8431-5512 (J.-S.W.); +86-592-2181851 (X.-K.Z.); +86-592-2195319 (X.-W.Y.)

† These authors contributed equally to this work.

Content

Figure S1. Chemical structures of compounds **7**–**28**.
Figure S2. HR-ESI-MS of citreoviridin J (**1**).
Figure S3. ^{1}H NMR spectrum (400 MHz, CD_3OD) of citreoviridin J (**1**).
Figure S4. ^{13}C NMR and DEPT spectra (100 MHz, CD_3OD) of citreoviridin J (**1**).
Figure S5. HMQC spectrum (400 MHz, CD_3OD) of citreoviridin J (**1**).
Figure S6. ^{1}H-^{1}H COSY spectrum (400 MHz, CD_3OD) of citreoviridin J (**1**).
Figure S7. HMBC spectrum (400 MHz, CD_3OD) of citreoviridin J (**1**).
Figure S8. NOESY spectrum of (400 MHz, CD_3OD) citreoviridin J (**1**).
Figure S9. HR-ESI-MS of citreoviridin K (**2**).
Figure S10. ^{1}H NMR spectrum (400 MHz, CD_3OD) of citreoviridin K (**2**).
Figure S11. ^{13}C NMR and DEPT (100 MHz, CD_3OD) of citreoviridin K (**2**).
Figure S12. HMQC spectrum (400 MHz, CD_3OD) of citreoviridin K (**2**).
Figure S13. ^{1}H-^{1}H COSY spectrum (400 MHz, CD_3OD) of citreoviridin K (**2**).
Figure S14. HMBC spectrum (400 MHz, CD_3OD) of citreoviridin K (**2**).
Figure S15. NOESY spectrum (400 MHz, CD_3OD) of citreoviridin K (**2**).
Figure S16. HR-ESI-MS of citreoviridin L (**3**).
Figure S17. ^{1}H NMR spectrum (400 MHz, CD_3OD) of citreoviridin L (**3**).
Figure S18. ^{13}C NMR and DEPT (100 MHz, CD_3OD) of citreoviridin L (**3**).
Figure S19. HMQC spectrum (400 MHz, CD_3OD) of citreoviridin L (**3**).
Figure S20. ^{1}H-^{1}H COSY spectrum (400 MHz, CD_3OD) of citreoviridin L (**3**).

Figure S21. HMBC spectrum (400 MHz, CD_3OD) of citreoviridin L (**3**).
Figure S22. NOESY spectrum (400 MHz, CD_3OD) of citreoviridin L (**3**).
Figure S23. HR-ESI-MS of citreoviridin M (**4**).
Figure S24. ^{1}H NMR spectrum (400 MHz, CD_3OD) of citreoviridin M (**4**).
Figure S25. ^{13}C NMR and DEPT (100 MHz, CD_3OD) of citreoviridin M (**4**).
Figure S26. HMQC spectrum (400 MHz, CD_3OD) of citreoviridin M (**4**).
Figure S27. ^{1}H-^{1}H COSY spectrum (400 MHz, CD_3OD) of citreoviridin M (**4**).
Figure S28. HMBC spectrum (400 MHz, CD_3OD) of citreoviridin M (**4**).
Figure S29. NOESY spectrum (400 MHz, CD_3OD) of citreoviridin M (**4**).
Figure S30. ^{1}H NMR spectrum (400 MHz, CD_3OD) of citreoviridins N and O (**5** and **6**).
Figure S31. Amplified ^{1}H NMR spectrum (400 MHz, CD_3OD) of citreoviridins N and O (**5** and **6**).
Figure S32. ^{13}C NMR and DEPT (100 MHz, CD_3OD) of citreoviridins N and O (**5** and **6**).
Figure S33. Amplified ^{13}C NMR spectrum (100 MHz, CD_3OD) of citreoviridins N and O (**5** and **6**).
Figure S34. HMQC spectrum (400 MHz, CD_3OD) of citreoviridins N and O (**5** and **6**).
Figure S35. ^{1}H-^{1}H COSY spectrum (400 MHz, CD_3OD) of citreoviridins N and O (**5** and **6**).
Figure S36. HMBC spectrum (400 MHz, CD_3OD) of citreoviridins N and O (**5** and **6**).
Figure S37. NOESY spectrum (400 MHz, CD_3OD) of citreoviridins N and O (**5** and **6**).
Figure S38. HR-ESI-MS of citreoviridin N (**5**).
Figure S39. ^{1}H NMR spectrum (400 MHz, CD_3OD) of citreoviridin N (**5**).
Figure S40. ^{1}H-^{1}H COSY spectrum (400 MHz, CD_3OD) of citreoviridin N (**5**).
Figure S41. NOESY spectrum (400 MHz, CD_3OD) of citreoviridin N (**5**).
Figure S42. HR-ESI-MS of citreoviridin O (**6**).
Figure S43. ^{1}H NMR spectrum (400 MHz, CD_3OD) of citreoviridin O (**6**).
Figure S44. ^{1}H-^{1}H COSY spectrum (400 MHz, CD_3OD) of citreoviridin O (**6**).
Figure S45. NOESY spectrum (400 MHz, CD_3OD) of citreoviridin O (**6**).
Figure S46. HR-ESI-MS of pyrenocine A (**7**).
Figure S47. ^{1}H NMR spectrum (400 MHz, CD_3OD) of pyrenocine A (**7**).
Figure S48. ^{13}C NMR spectrum (100 MHz, CD_3OD) of pyrenocine A (**7**).
Figure S49. HR-ESI-MS of terrein (**14**).
Figure S50. ^{1}H NMR spectrum (400 MHz, CD_3OD) of terrein (**14**).
Figure S51. ^{13}C NMR spectrum (100 MHz, CD_3OD) of terrein (**14**).
Figure S52. HR-ESI-MS of citreoviridin (**20**).
Figure S53. ^{1}H NMR spectrum (400 MHz, CD_3OD) of citreoviridin (**20**).
Figure S54. ^{13}C NMR spectrum (100 MHz, CD_3OD) of citreoviridin (**20**).

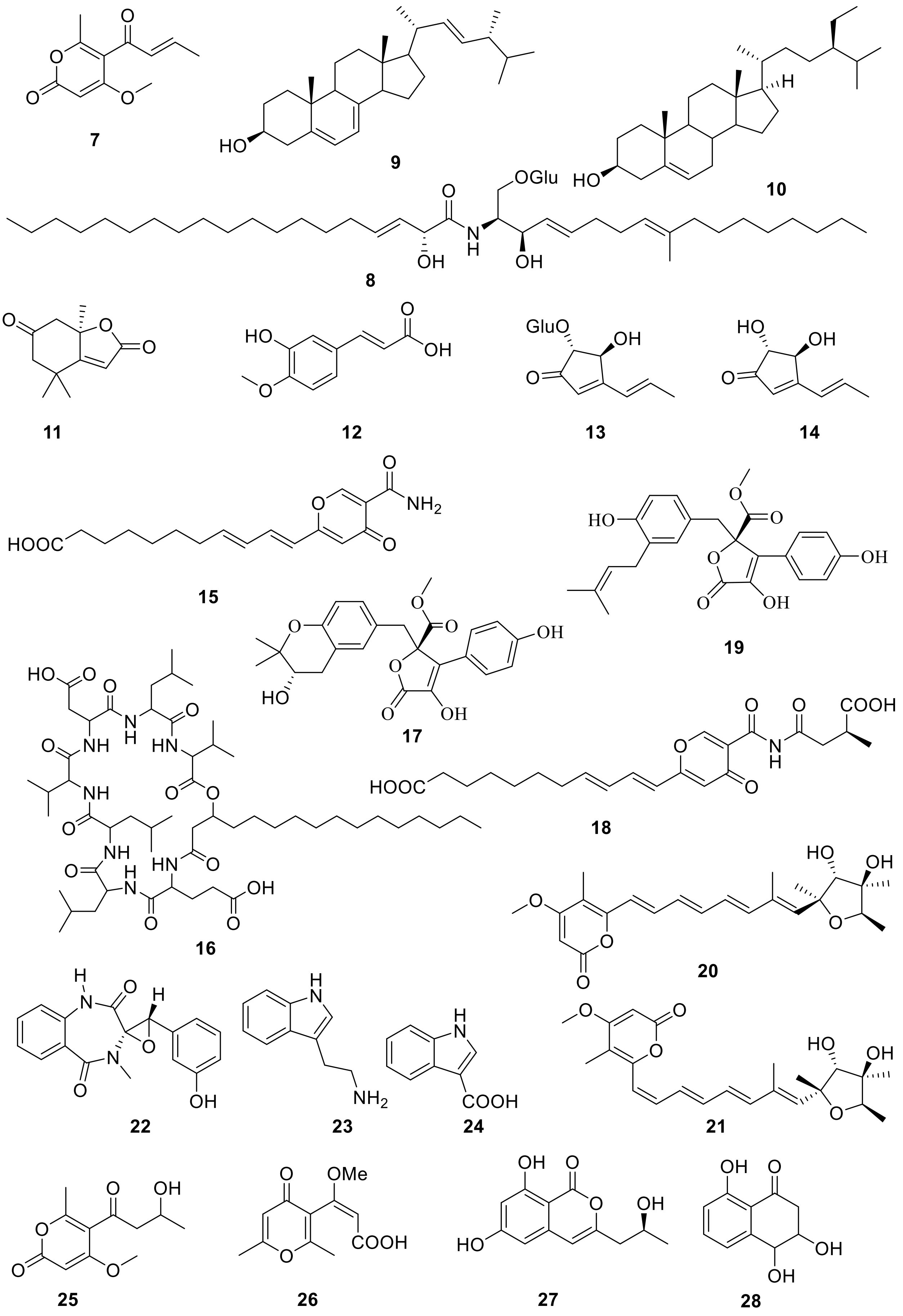

Figure S1 Chemical structures of compounds **7**–**28**.

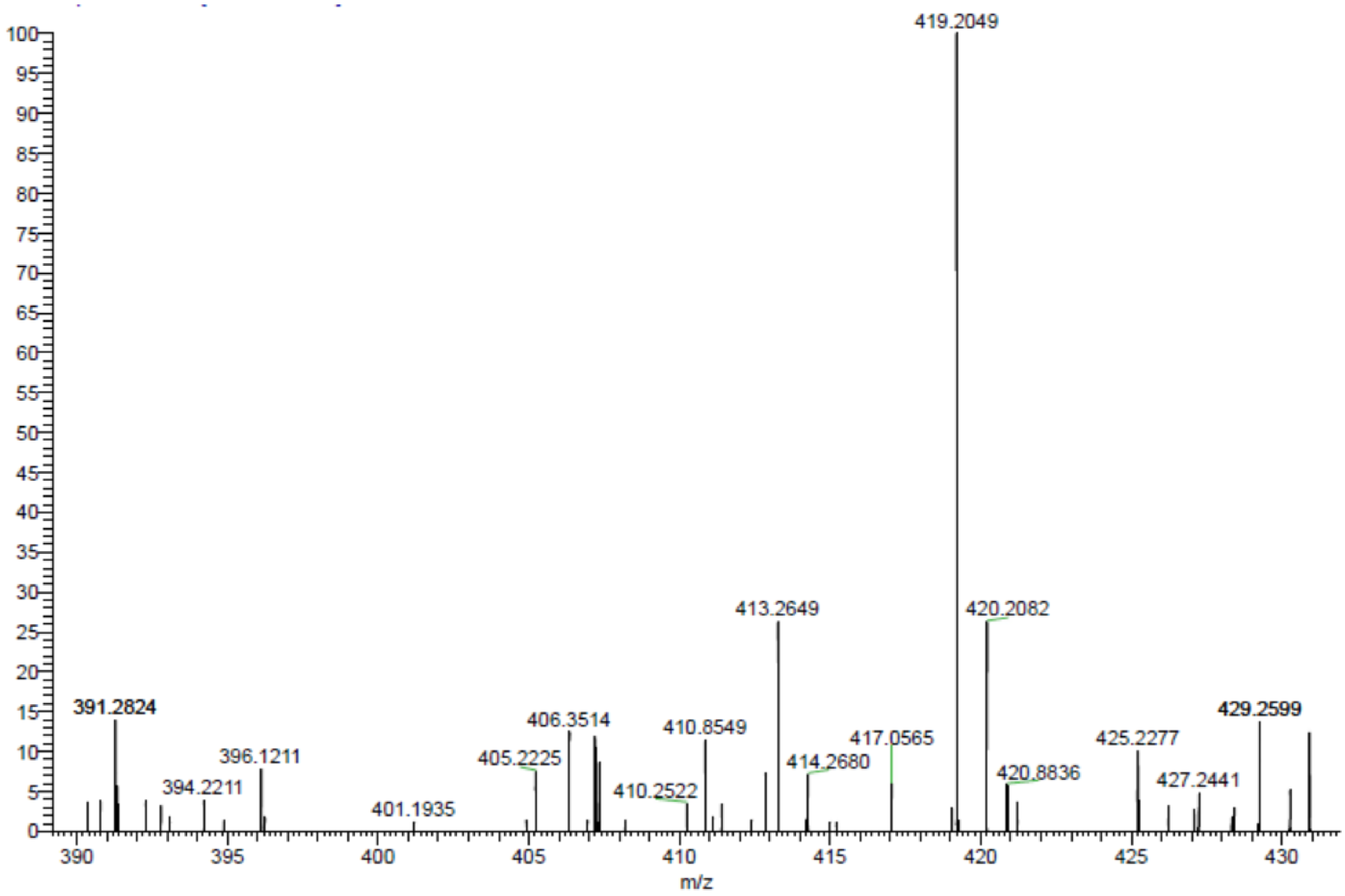

Figure S2. HR-ESI-MS of citreoviridin J (**1**)

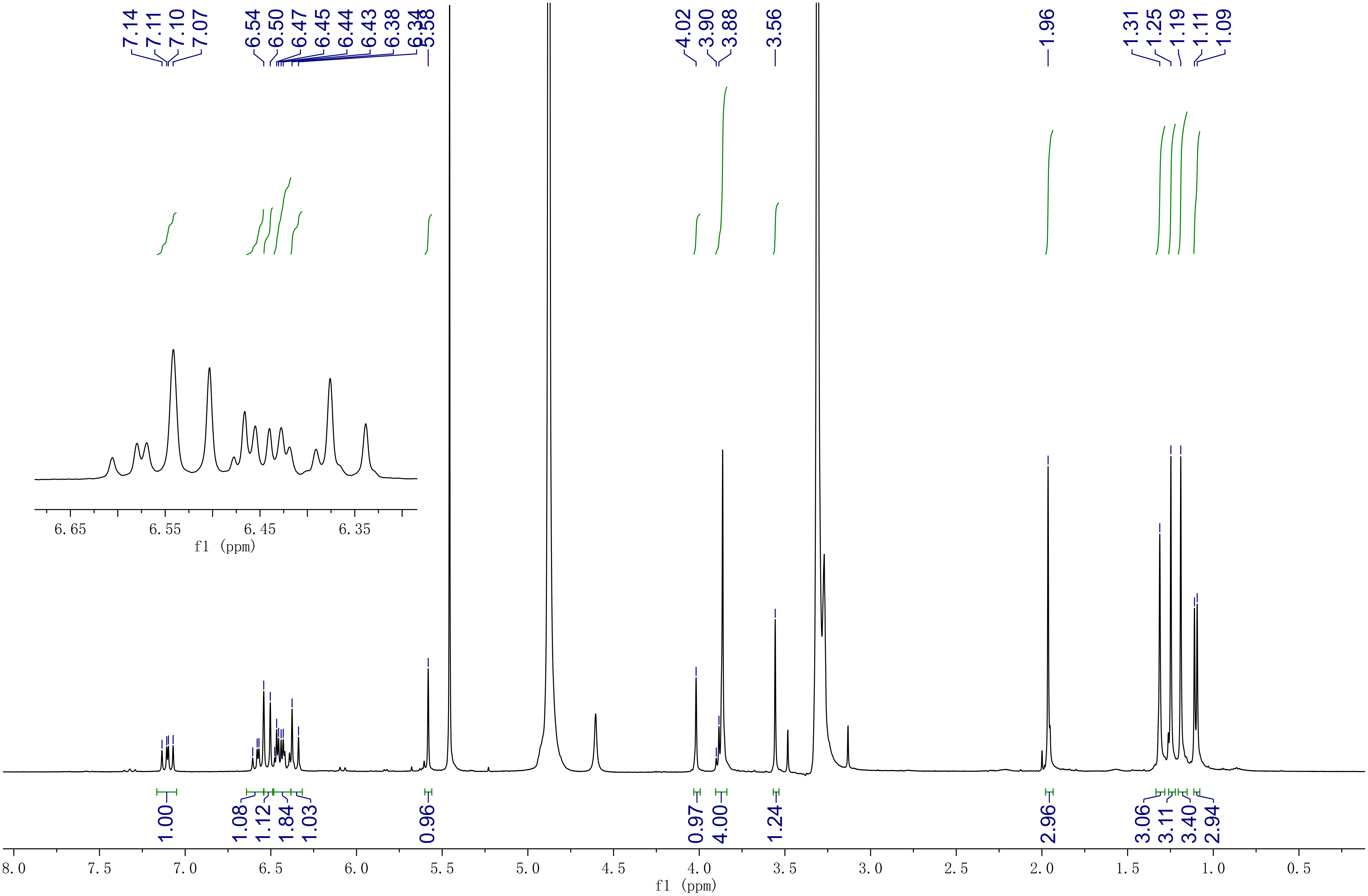

Figure S3. ^{1}H NMR spectrum (400 MHz, CD_3OD) of citreoviridin J (**1**)

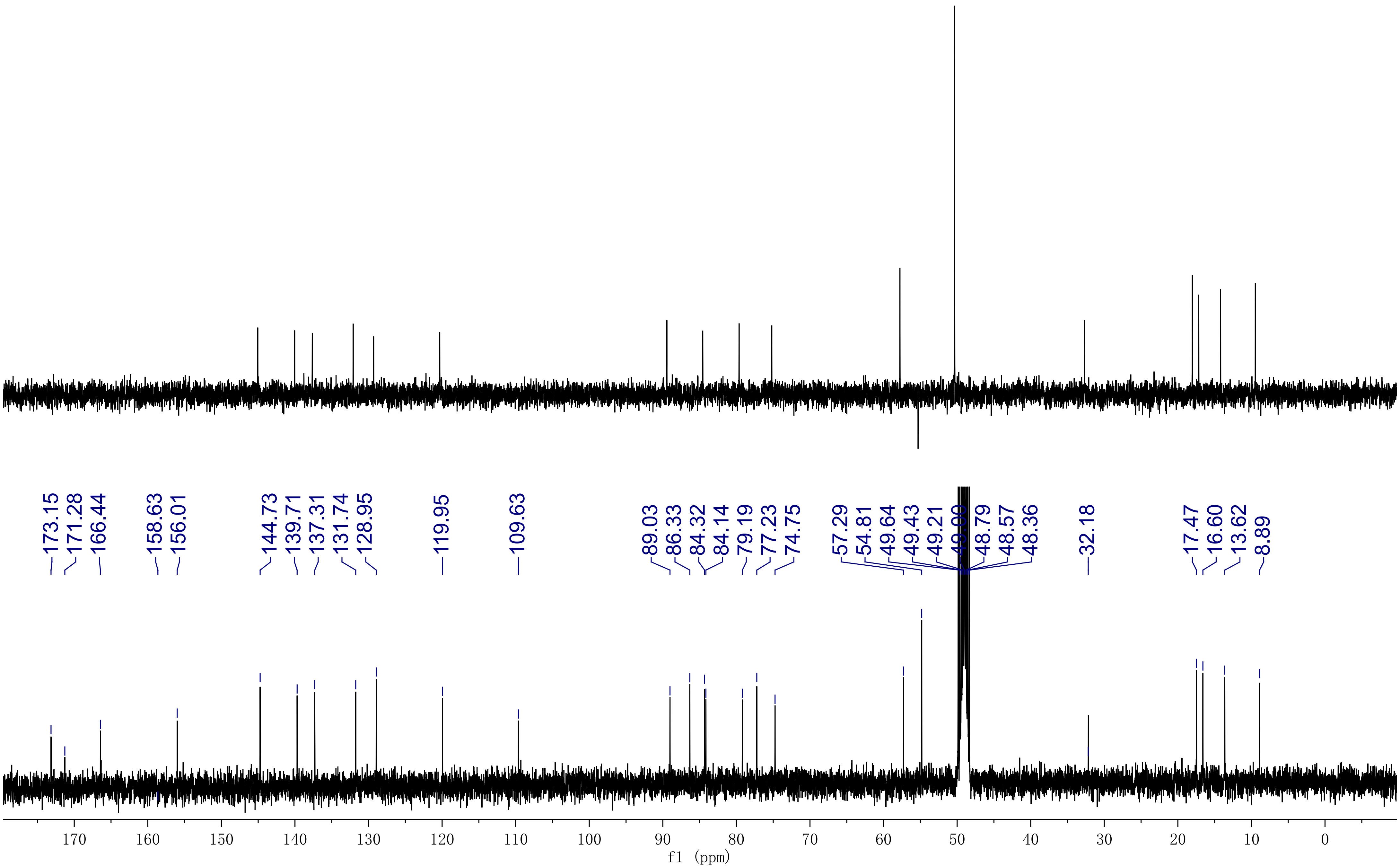

Figure S4. ^{13}C NMR and DEPT spectra (100 MHz, CD_3OD) of citreoviridin J (**1**)

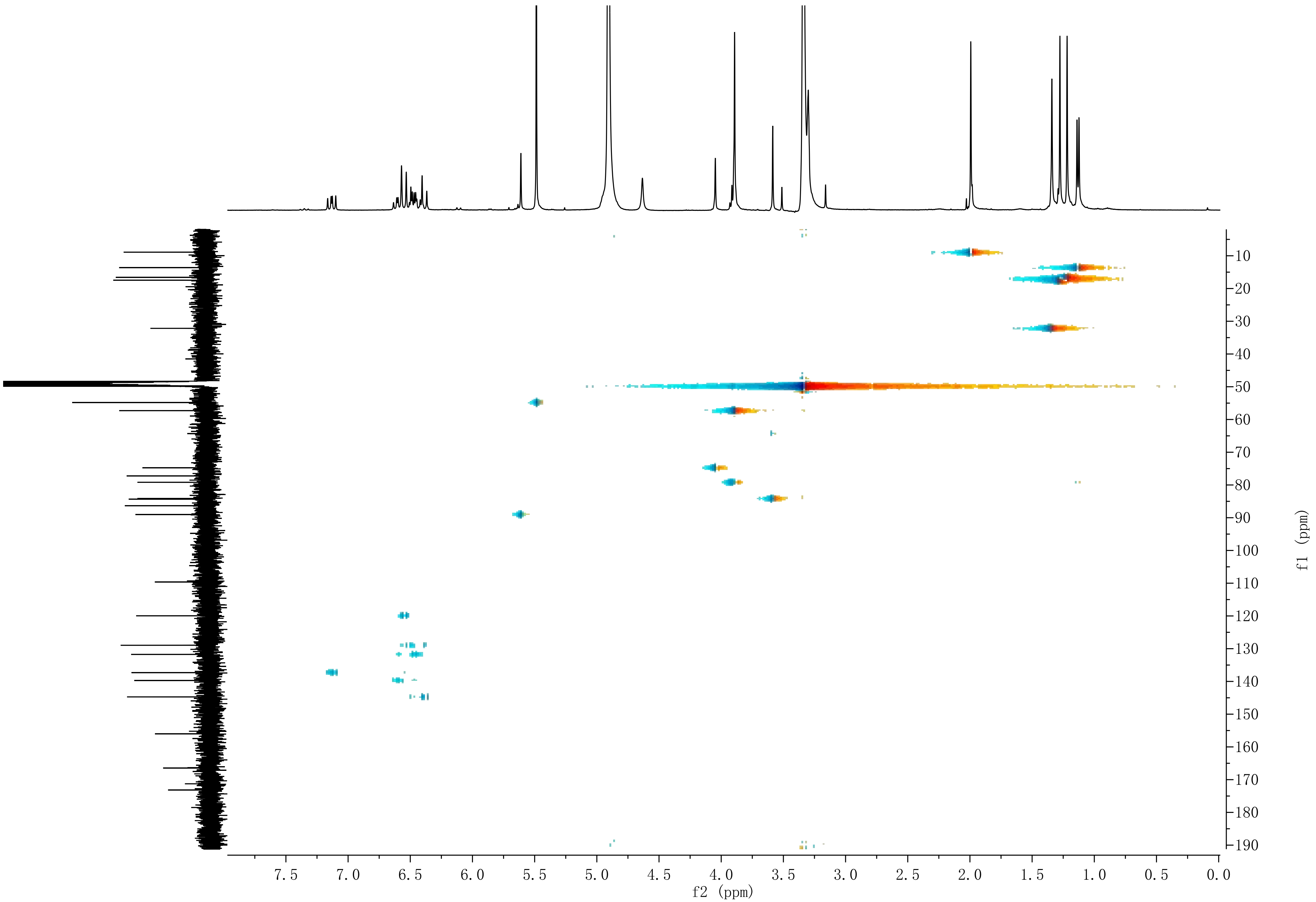

Figure S5. HMQC spectrum (400 MHz, CD_3OD) of citreoviridin J (**1**)

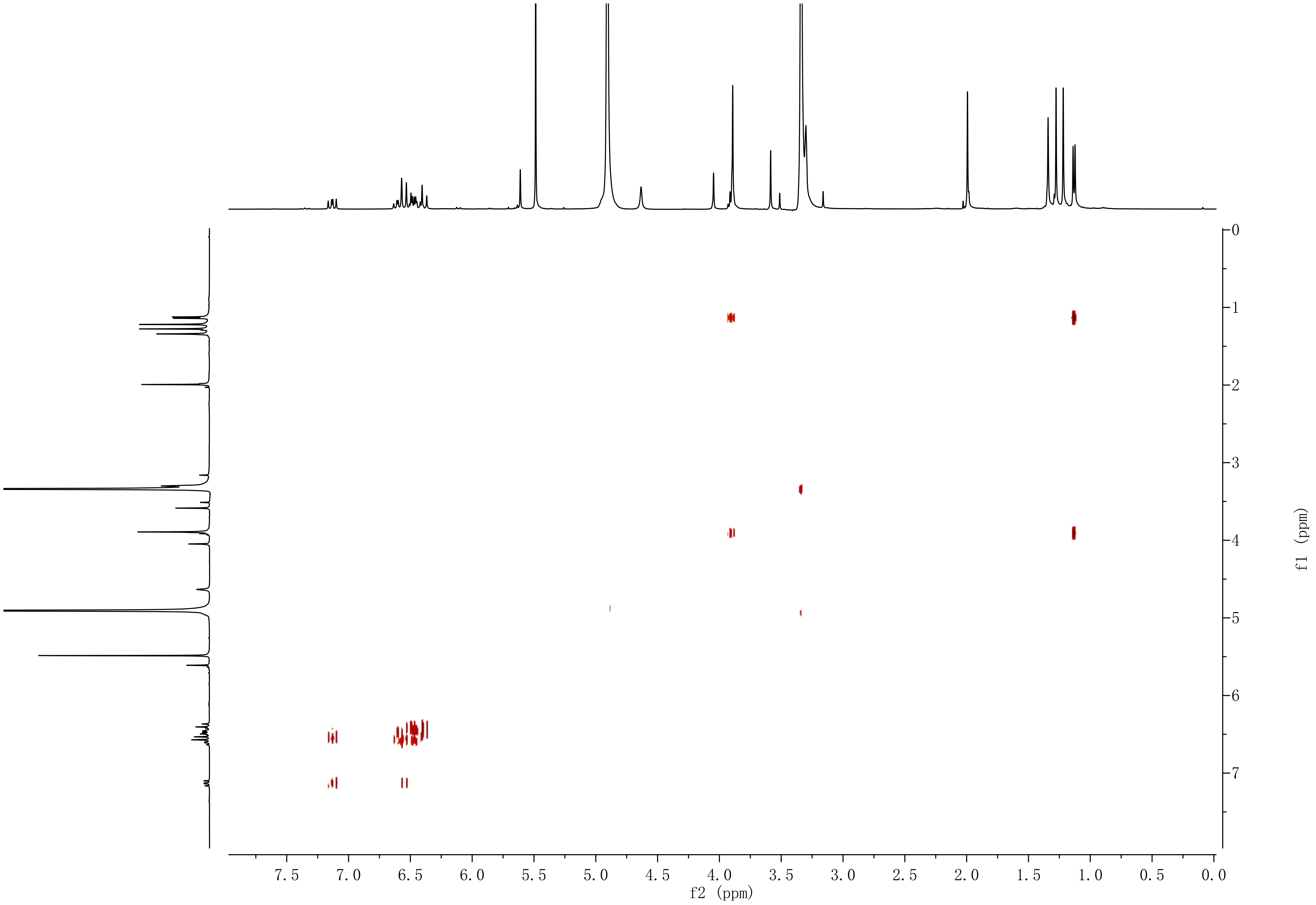

Figure S6. ^{1}H-^{1}H COSY spectrum (400 MHz, CD_3OD) of citreoviridin J (**1**)

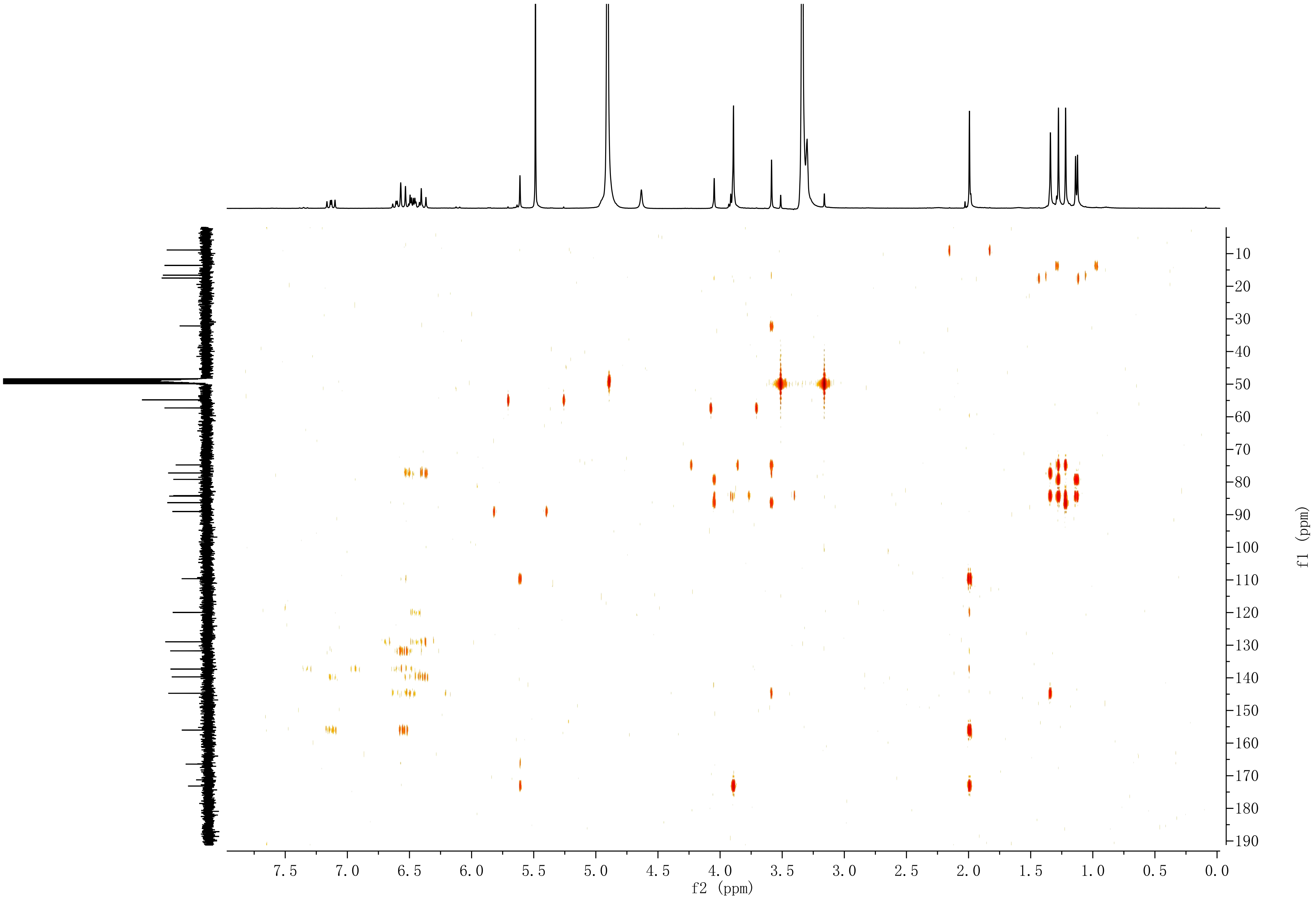

Figure S7. HMBC spectrum (400 MHz, CD_3OD) of citreoviridin J (**1**)

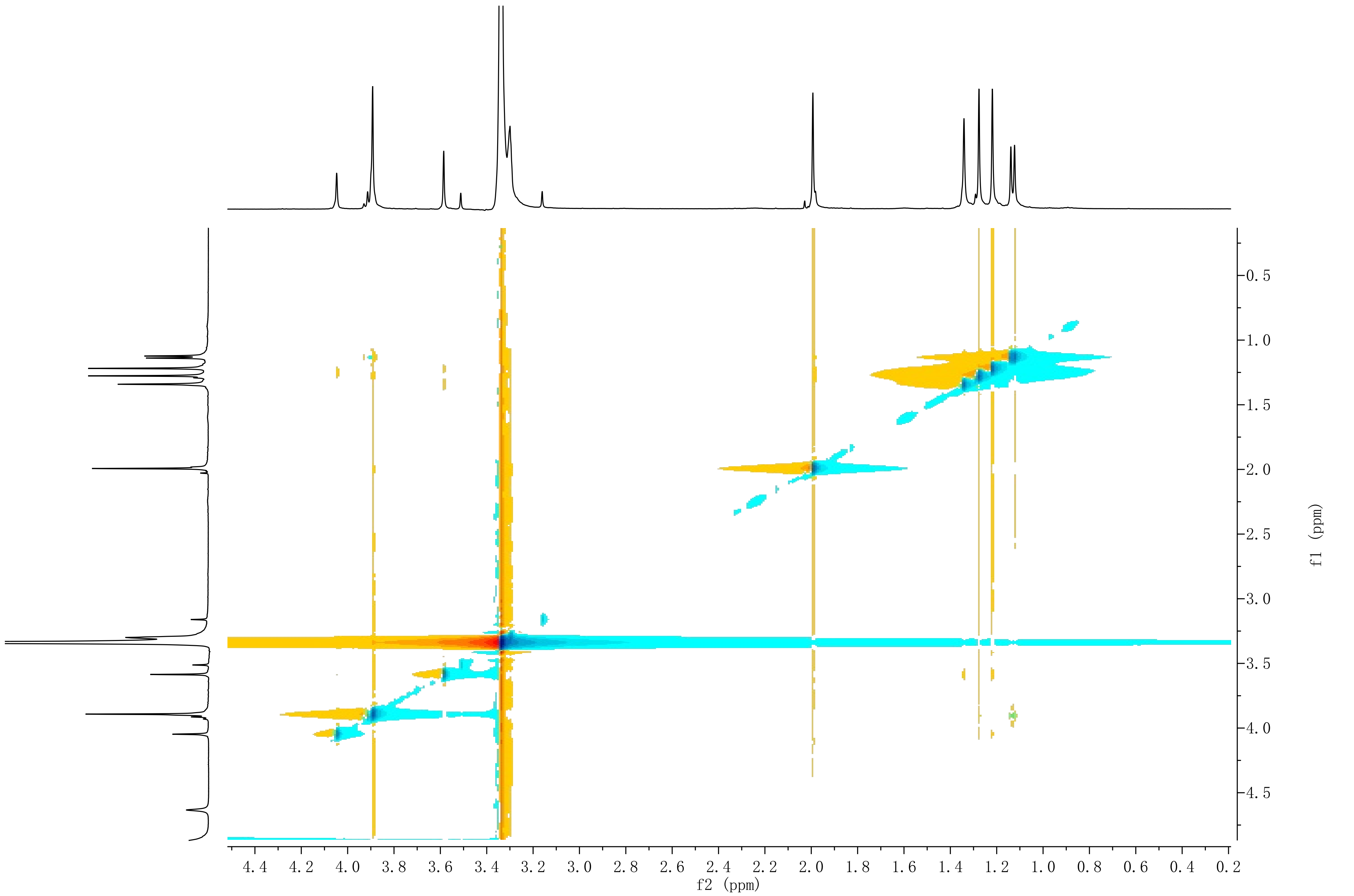

Figure S8. NOESY spectrum of (400 MHz, CD_3OD) citreoviridin J (**1**)

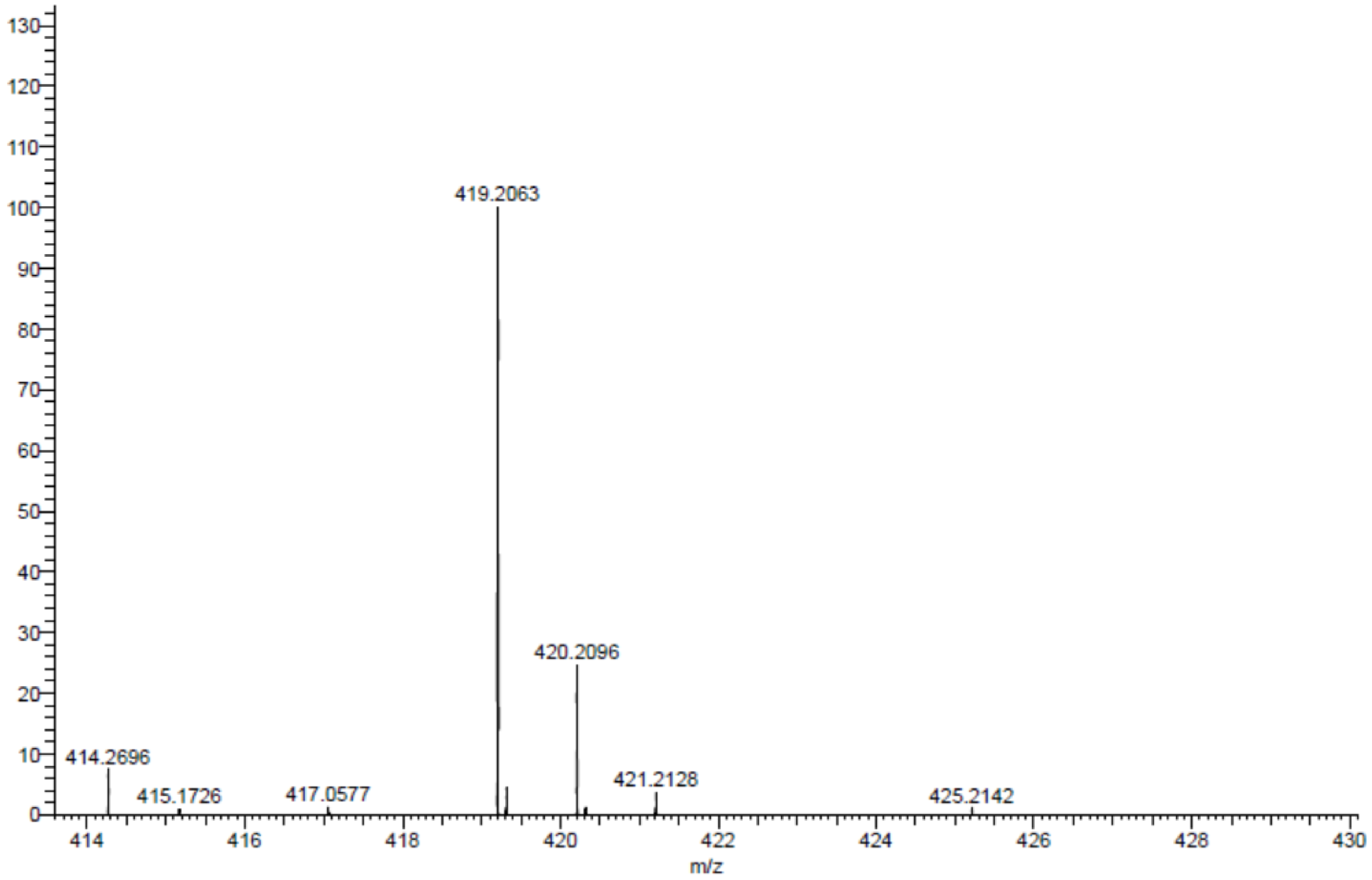

Figure S9. HR-ESI-MS of citreoviridin K (**2**)

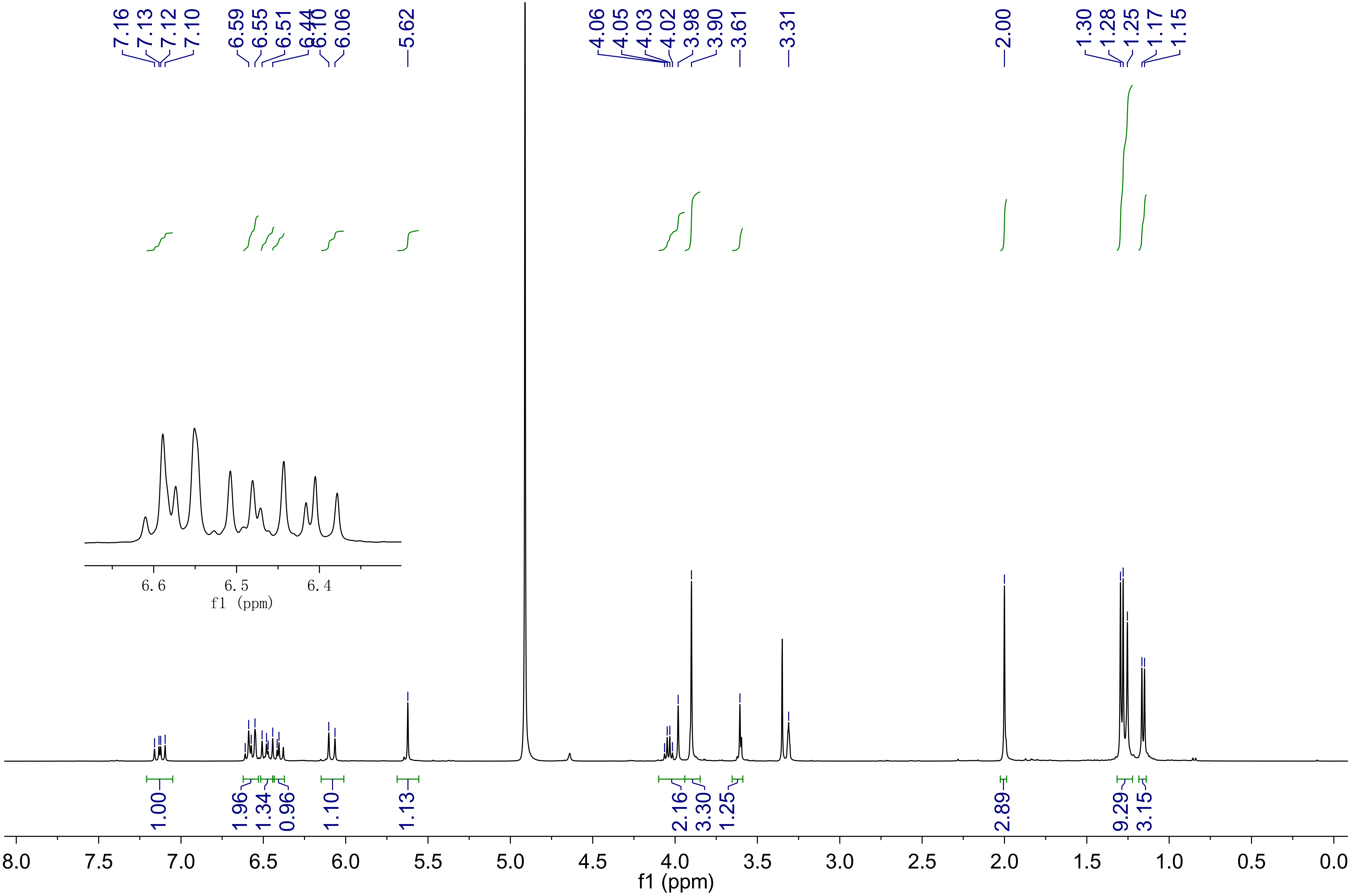

Figure S10. ^{1}H NMR spectrum (400 MHz, CD_3OD) of citreoviridin K (**2**)

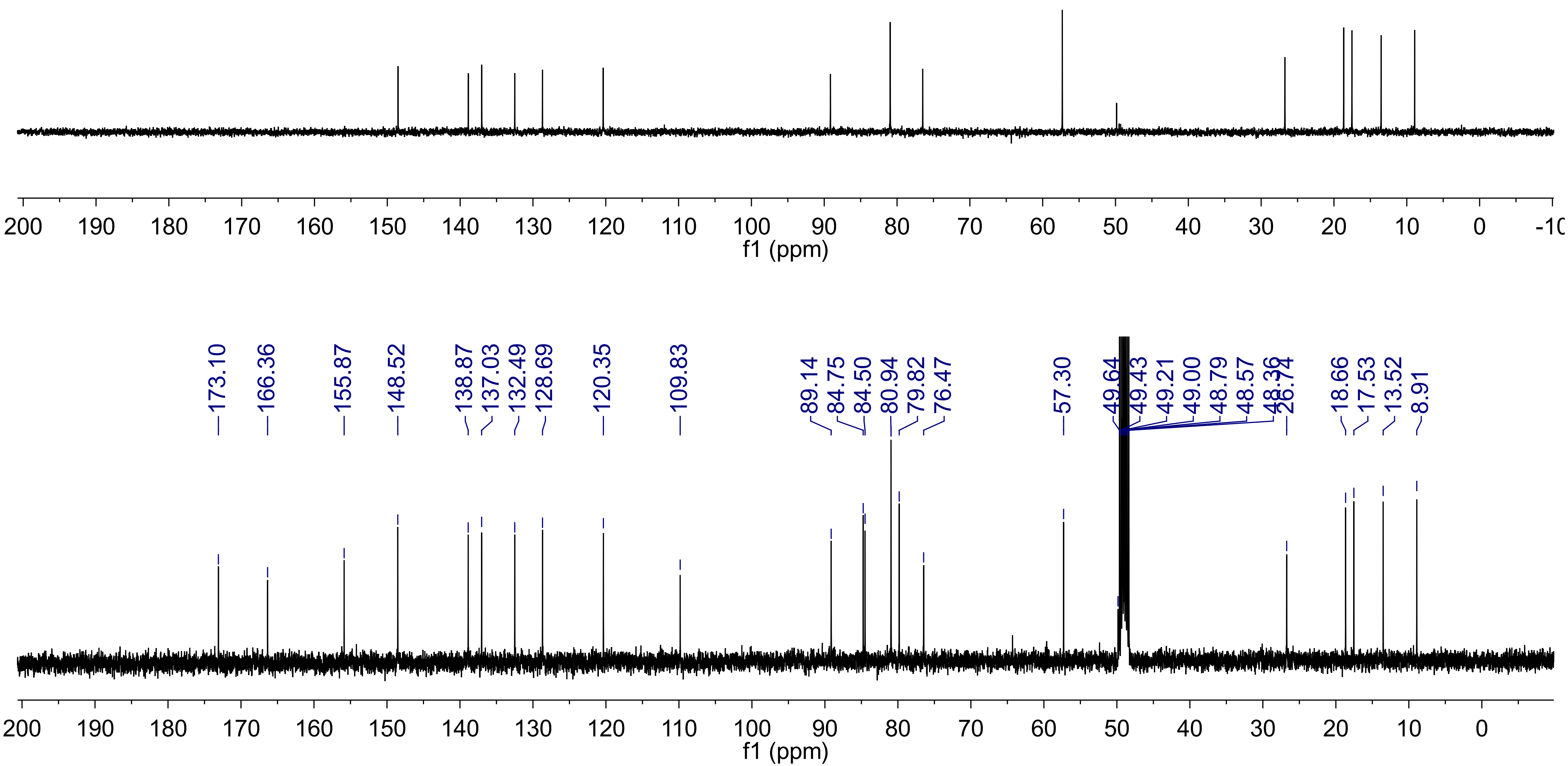

Figure S11. ^{13}C NMR and DEPT (100 MHz, CD_3OD) of citreoviridin K (**2**)

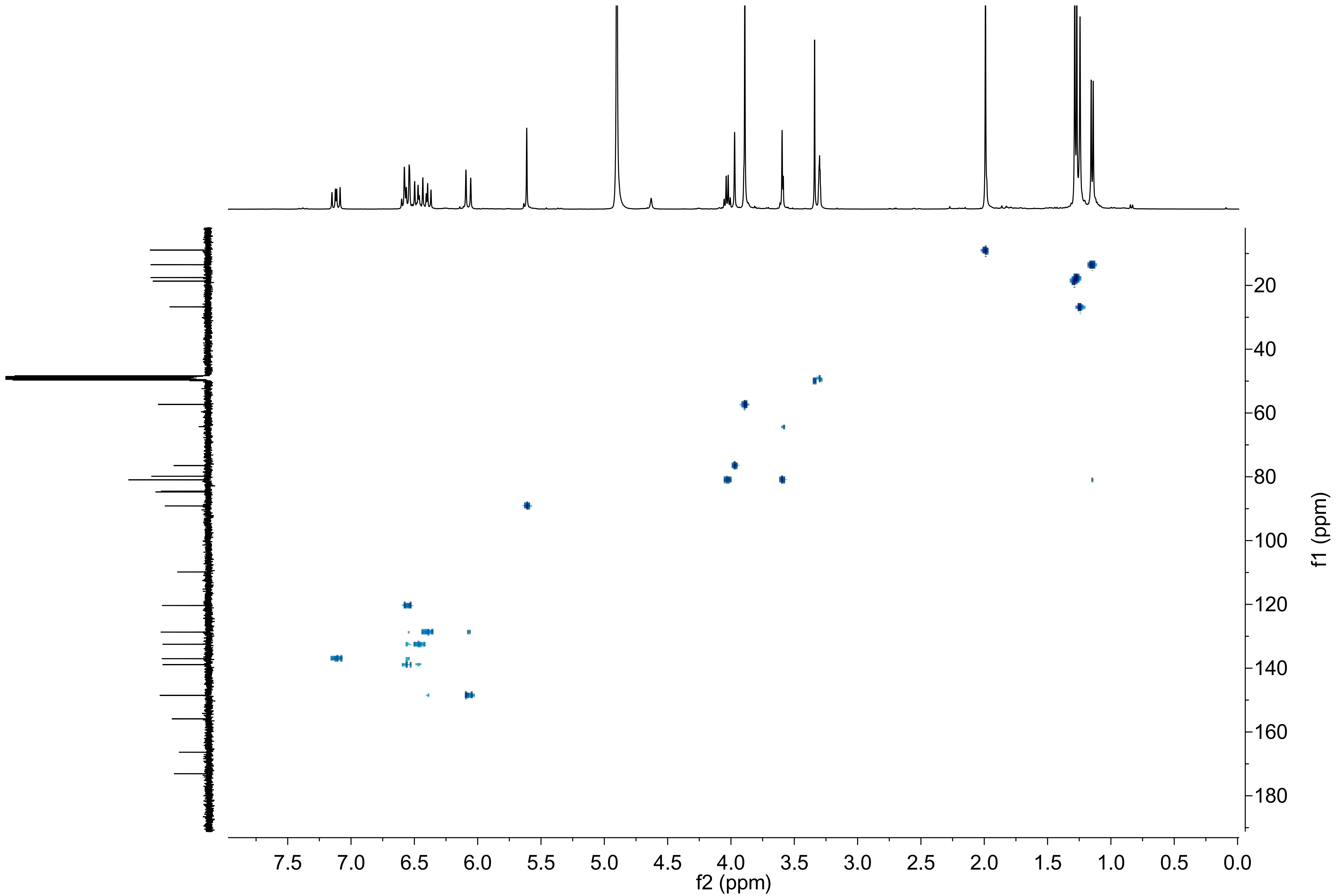

Figure S12. HMQC spectrum (400 MHz, CD_3OD) of citreoviridin K (**2**)

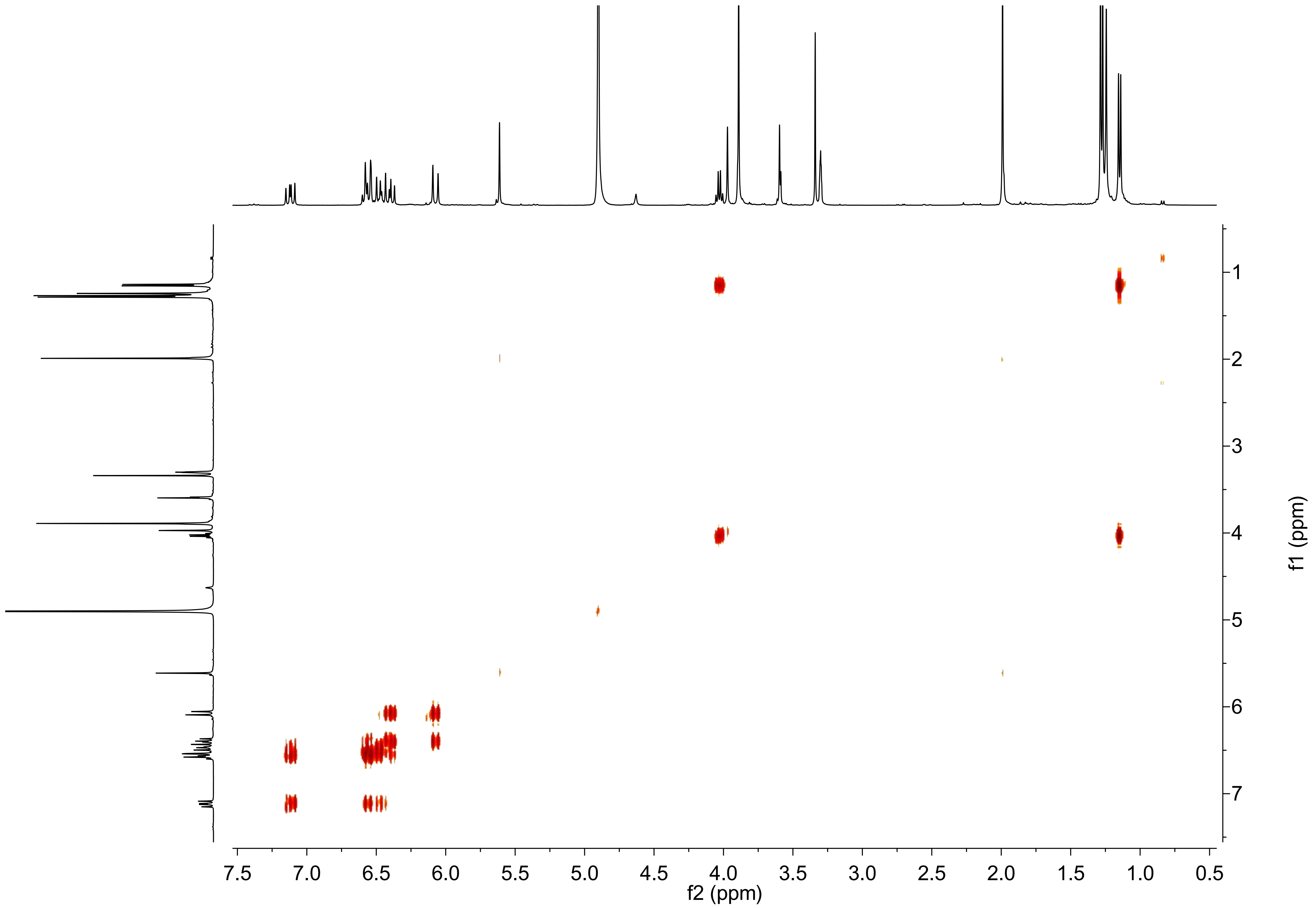

Figure S13. ^{1}H-^{1}H COSY spectrum (400 MHz, CD_3OD) of citreoviridin K (**2**)

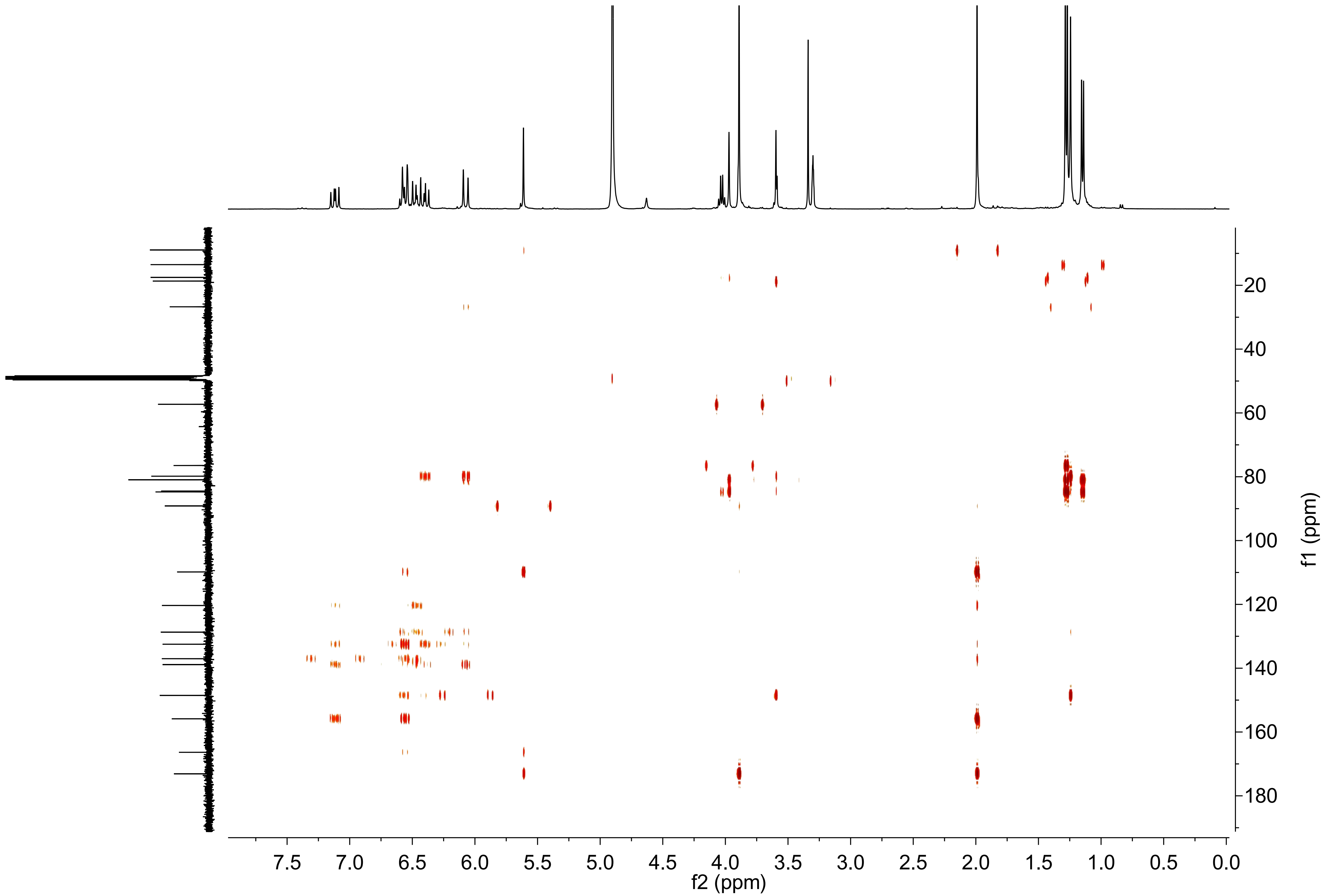

Figure S14. HMBC spectrum (400 MHz, CD_3OD) of citreoviridin K (**2**)

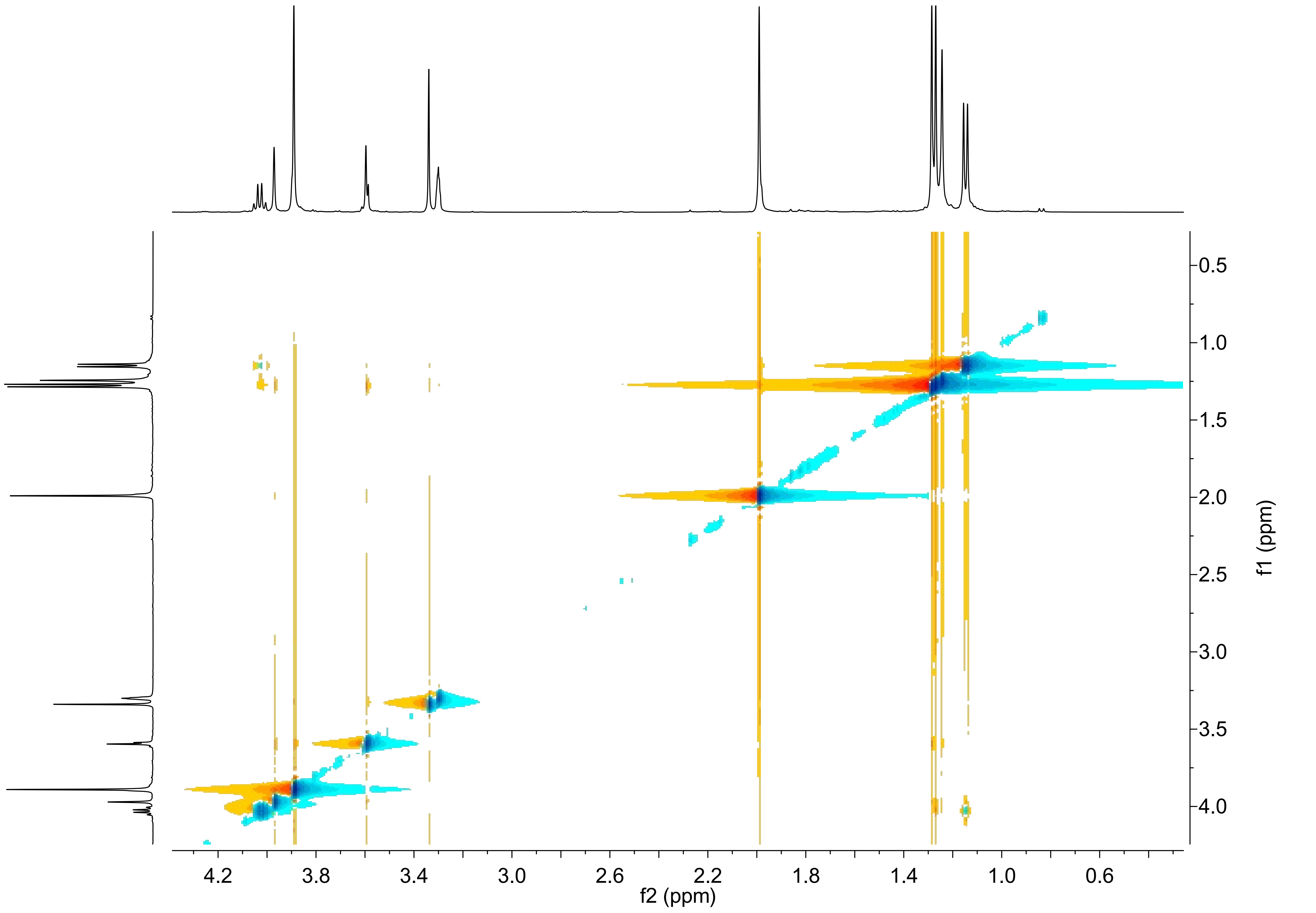

Figure S15. NOESY spectrum (400 MHz, CD_3OD) of citreoviridin K (**2**)

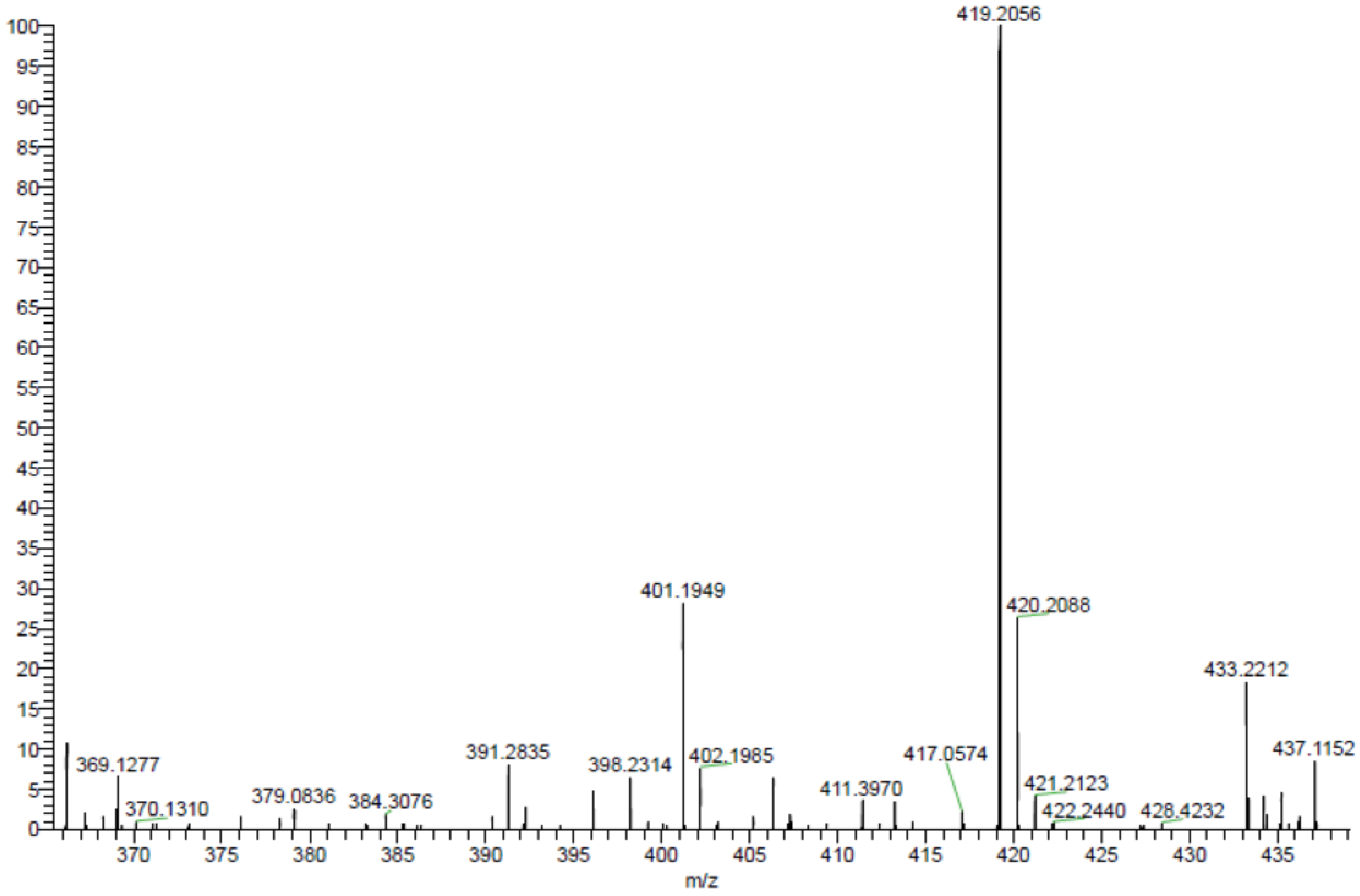

Figure S16. HR-ESI-MS of citreoviridin L (**3**)

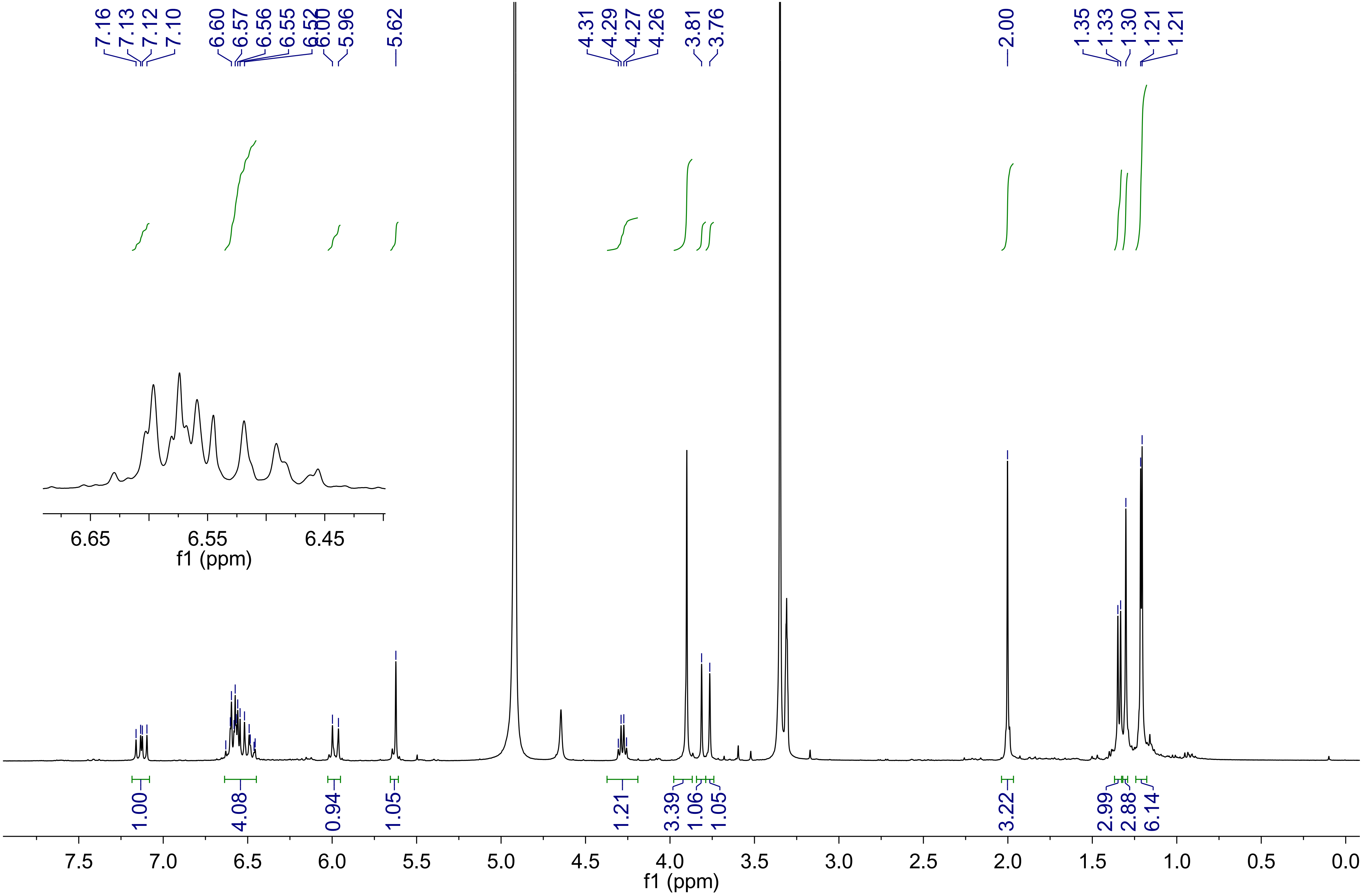

Figure S17. ^{1}H NMR spectrum (400 MHz, CD_3OD) of citreoviridin L (**3**)

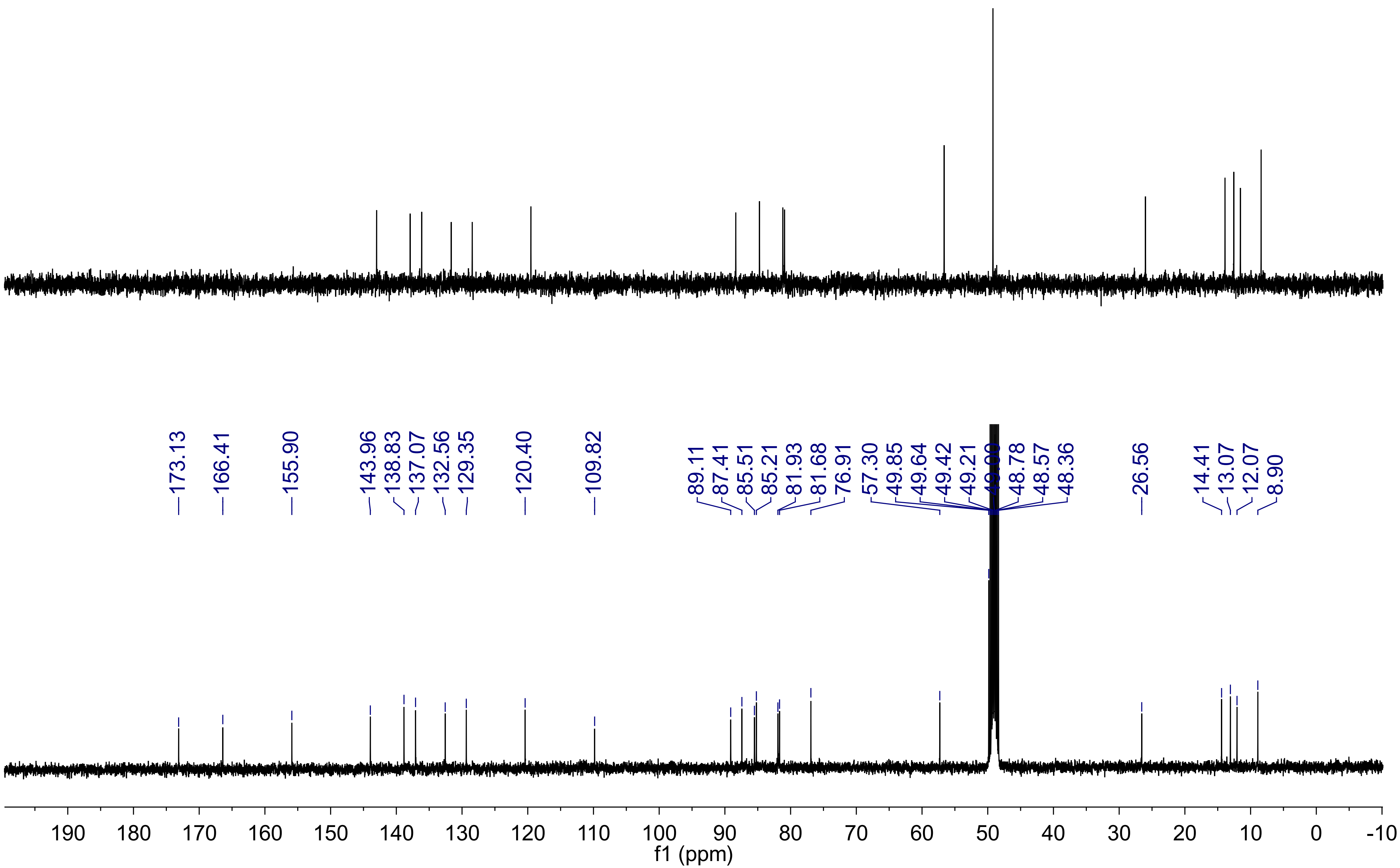

Figure S18. ^{13}C NMR and DEPT (100 MHz, CD_3OD) of citreoviridin L (**3**)

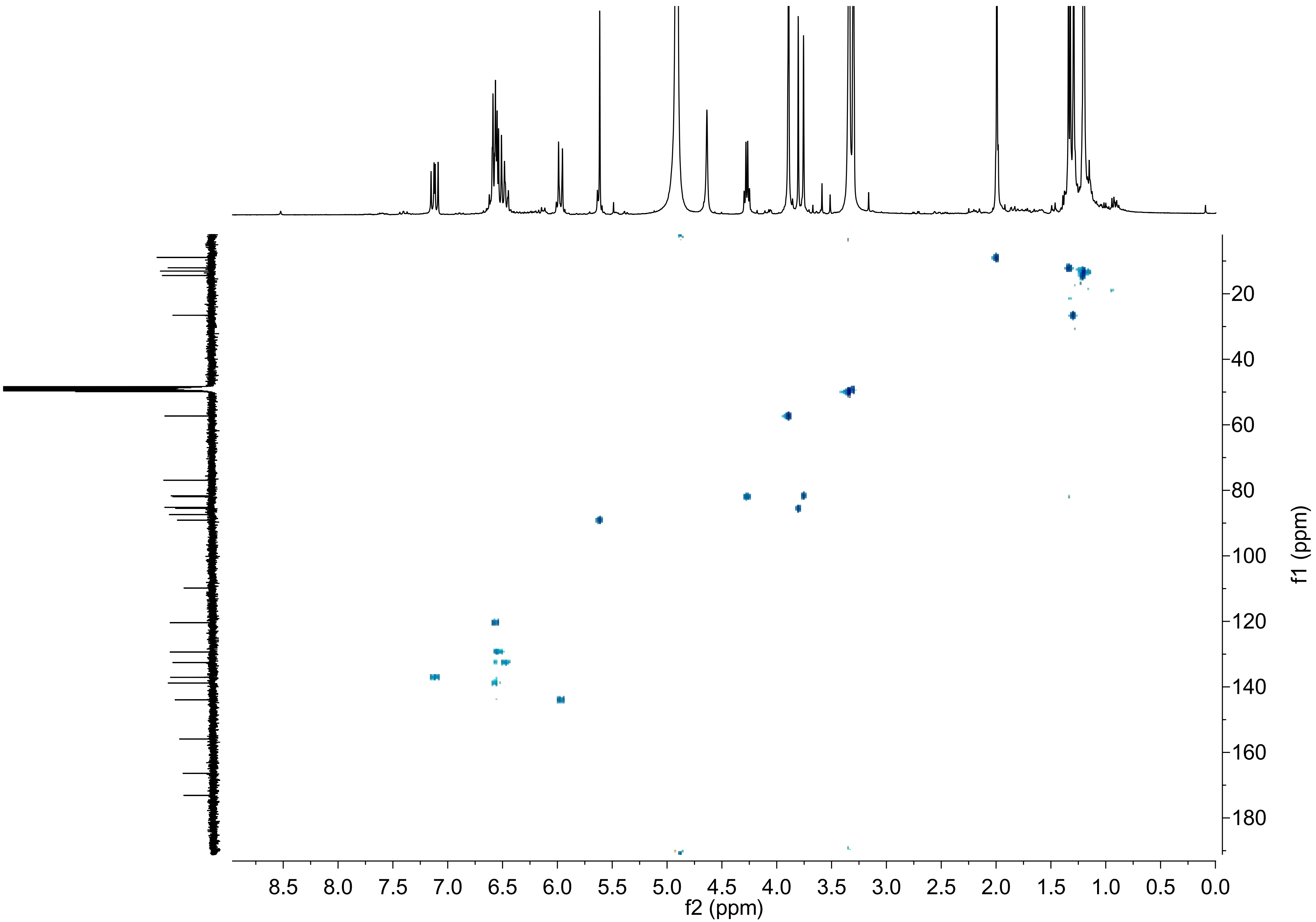

Figure S19. HMQC spectrum (400 MHz, CD_3OD) of citreoviridin L (**3**)

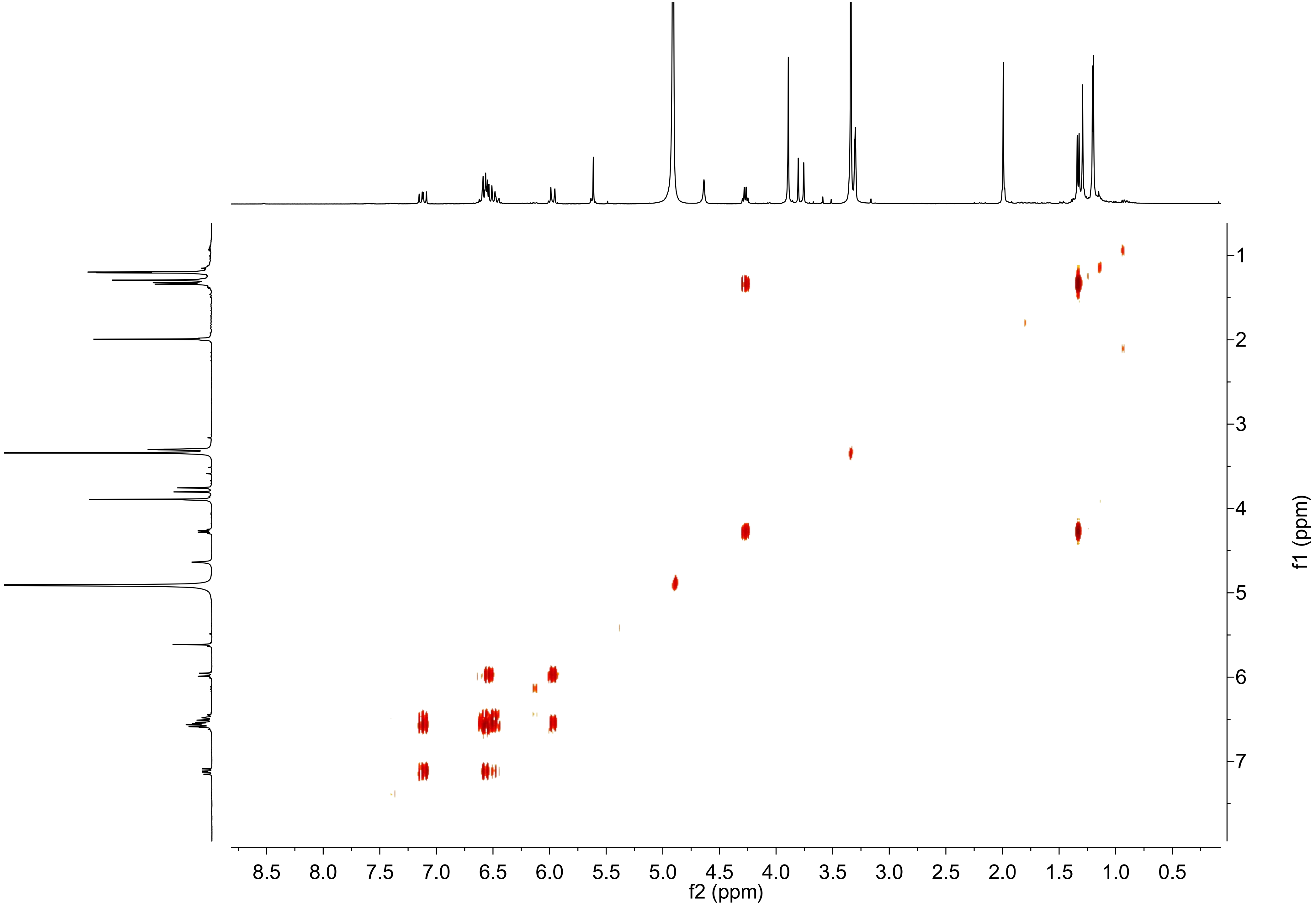

Figure S20. ^{1}H-^{1}H COSY spectrum (400 MHz, CD_3OD) of citreoviridin L (**3**)

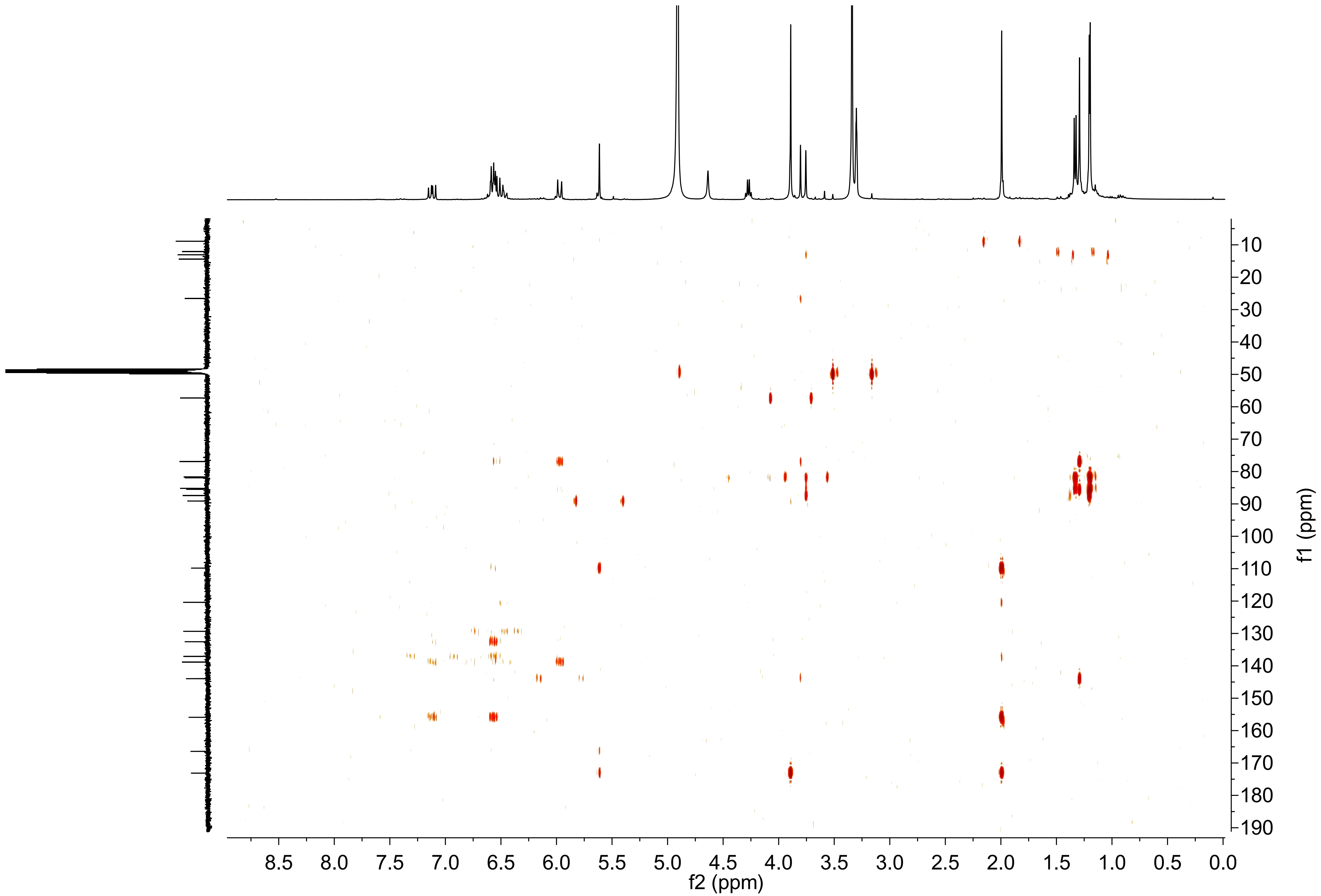

Figure S21. HMBC spectrum (400 MHz, CD_3OD) of citreoviridin L (**3**)

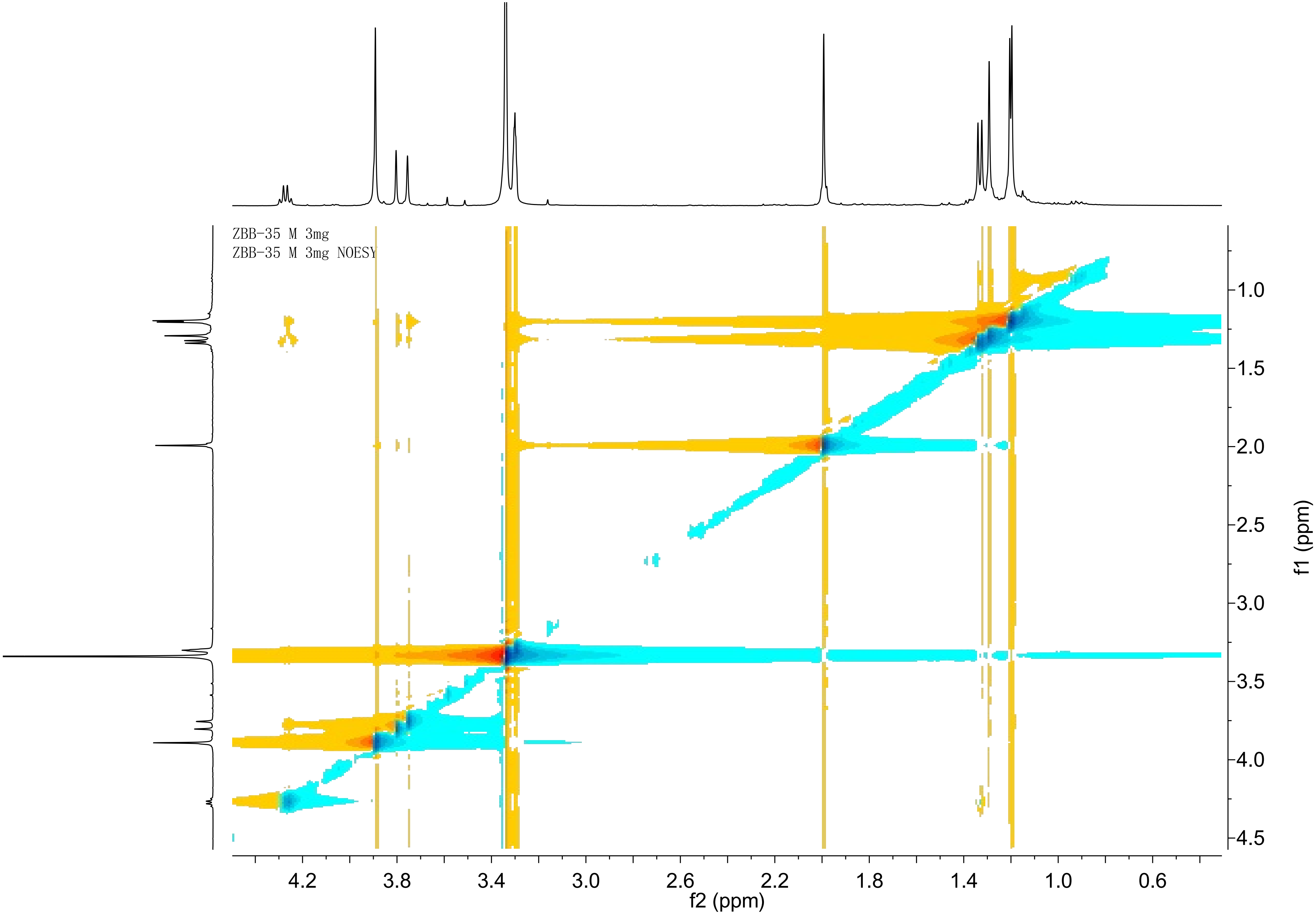

Figure S22. NOESY spectrum (400 MHz, CD_3OD) of citreoviridin L (**3**)

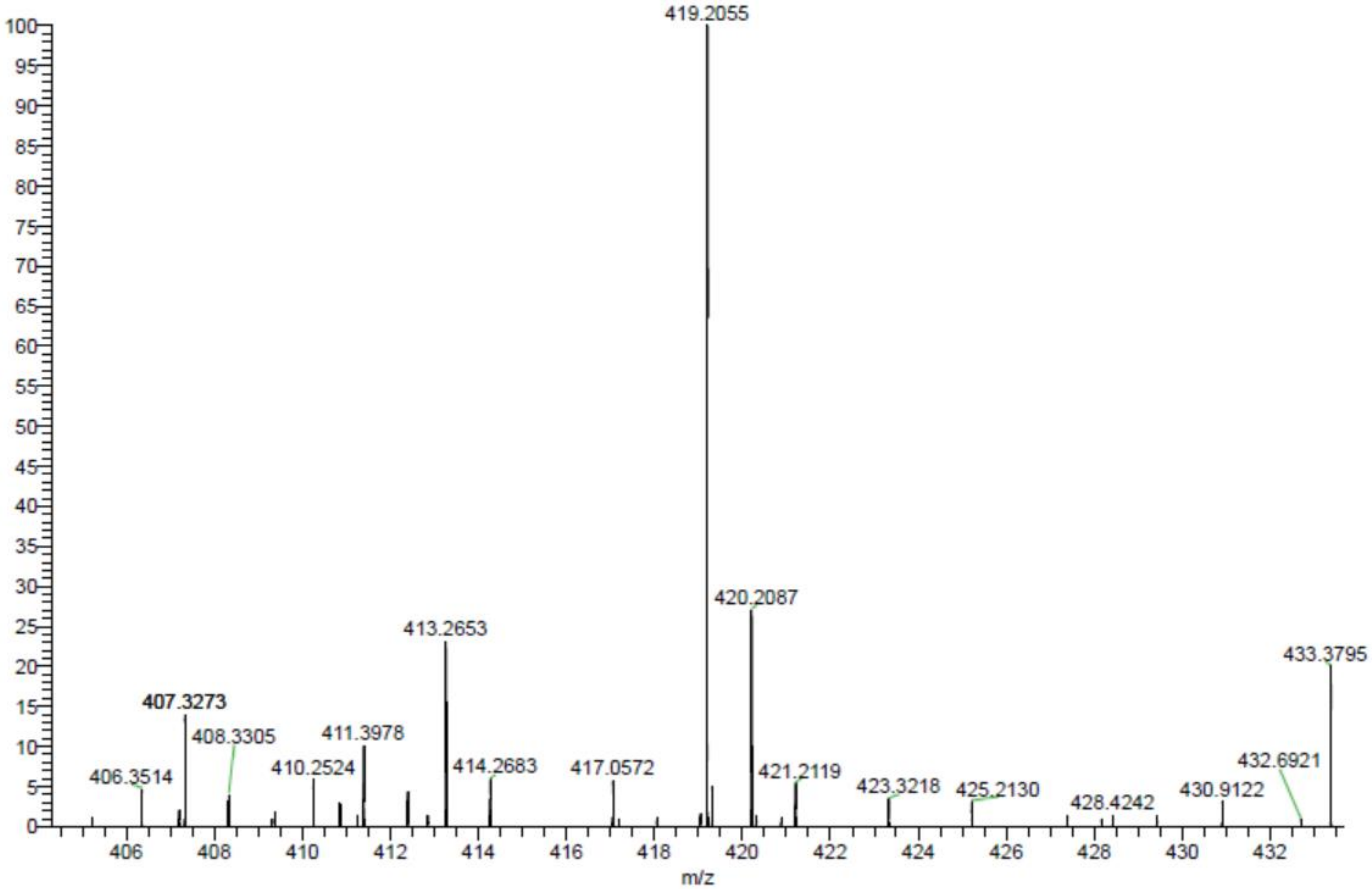

Figure S23. HR-ESI-MS of citreoviridin M (**4**)

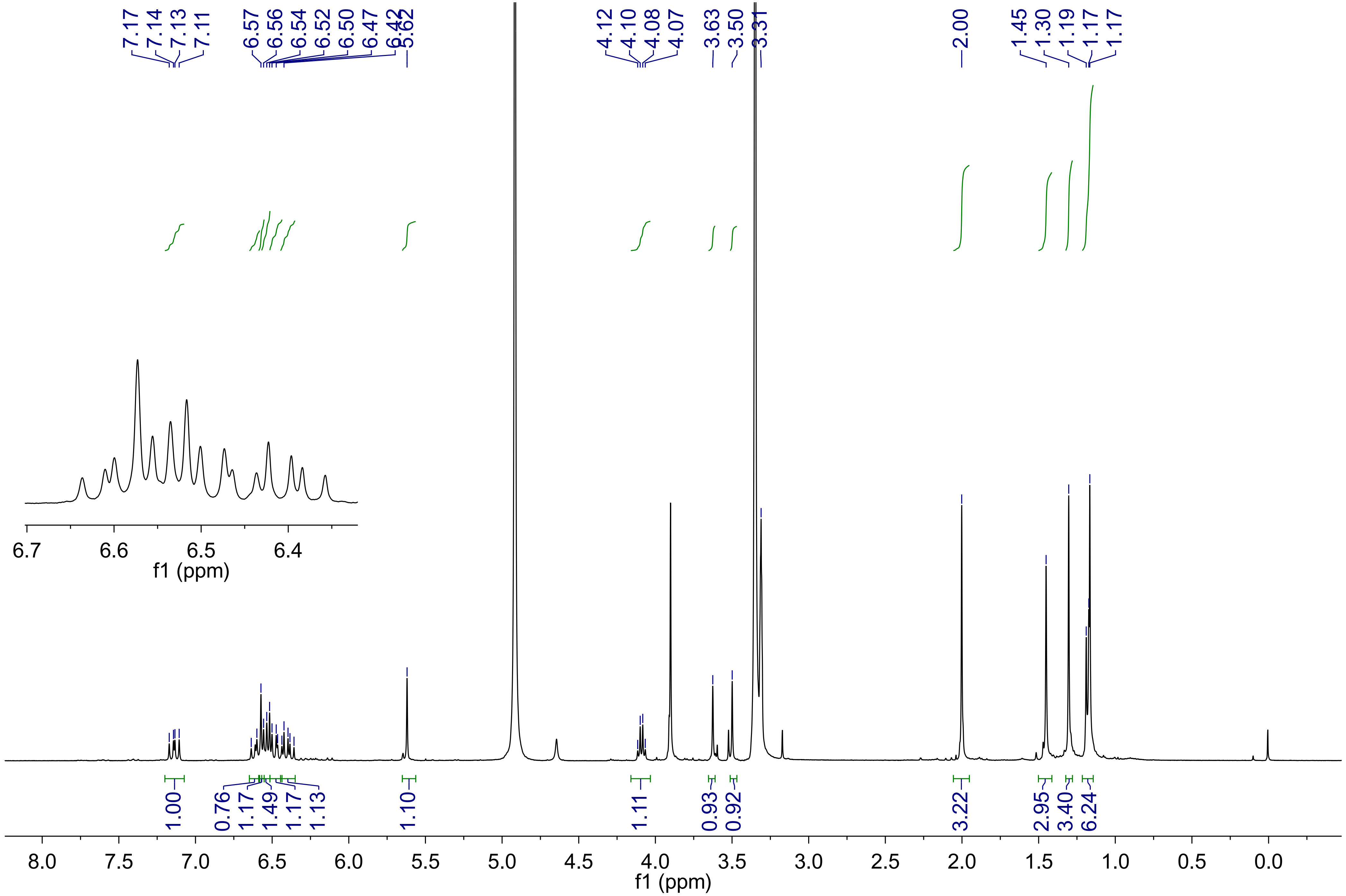

Figure S24. ^{1}H NMR spectrum (400 MHz, CD_3OD) of citreoviridin M (**4**)

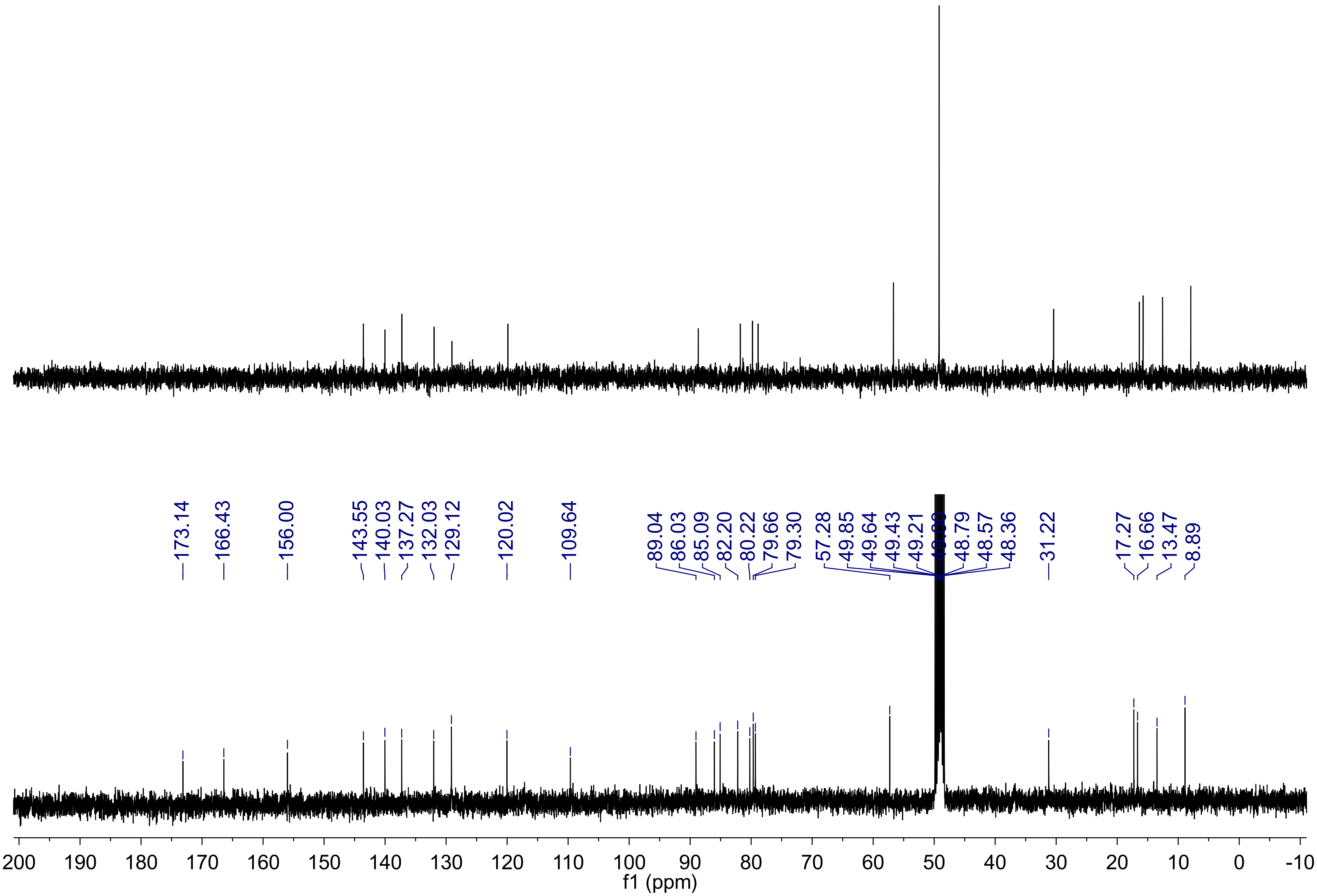

Figure S25. ^{13}C NMR and DEPT (100 MHz, CD_3OD) of citreoviridin M (**4**)

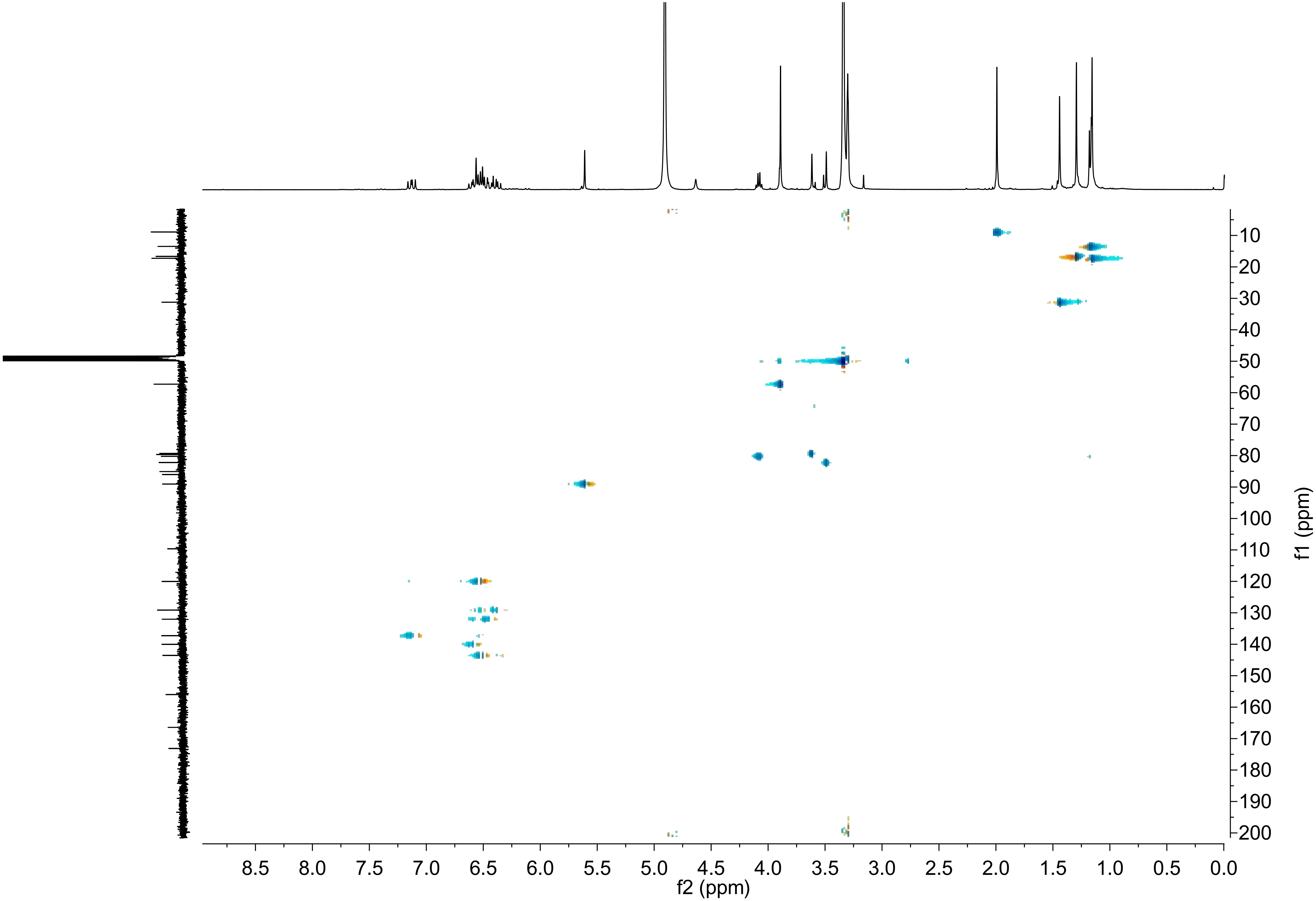

Figure S26. HMQC spectrum (400 MHz, CD_3OD) of citreoviridin M (**4**)

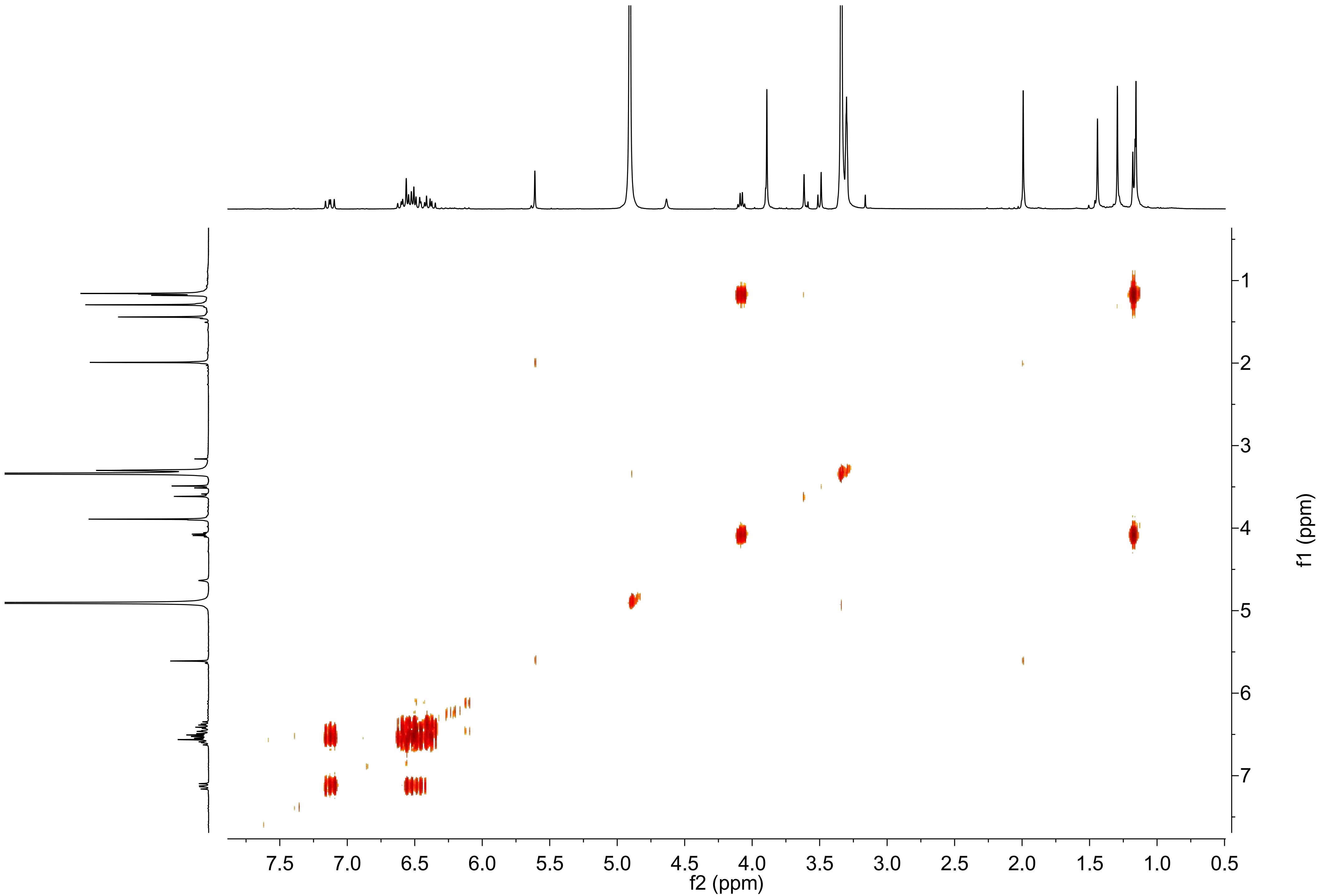

Figure S27. ^{1}H-^{1}H COSY spectrum (400 MHz, CD_3OD) of citreoviridin M (**4**)

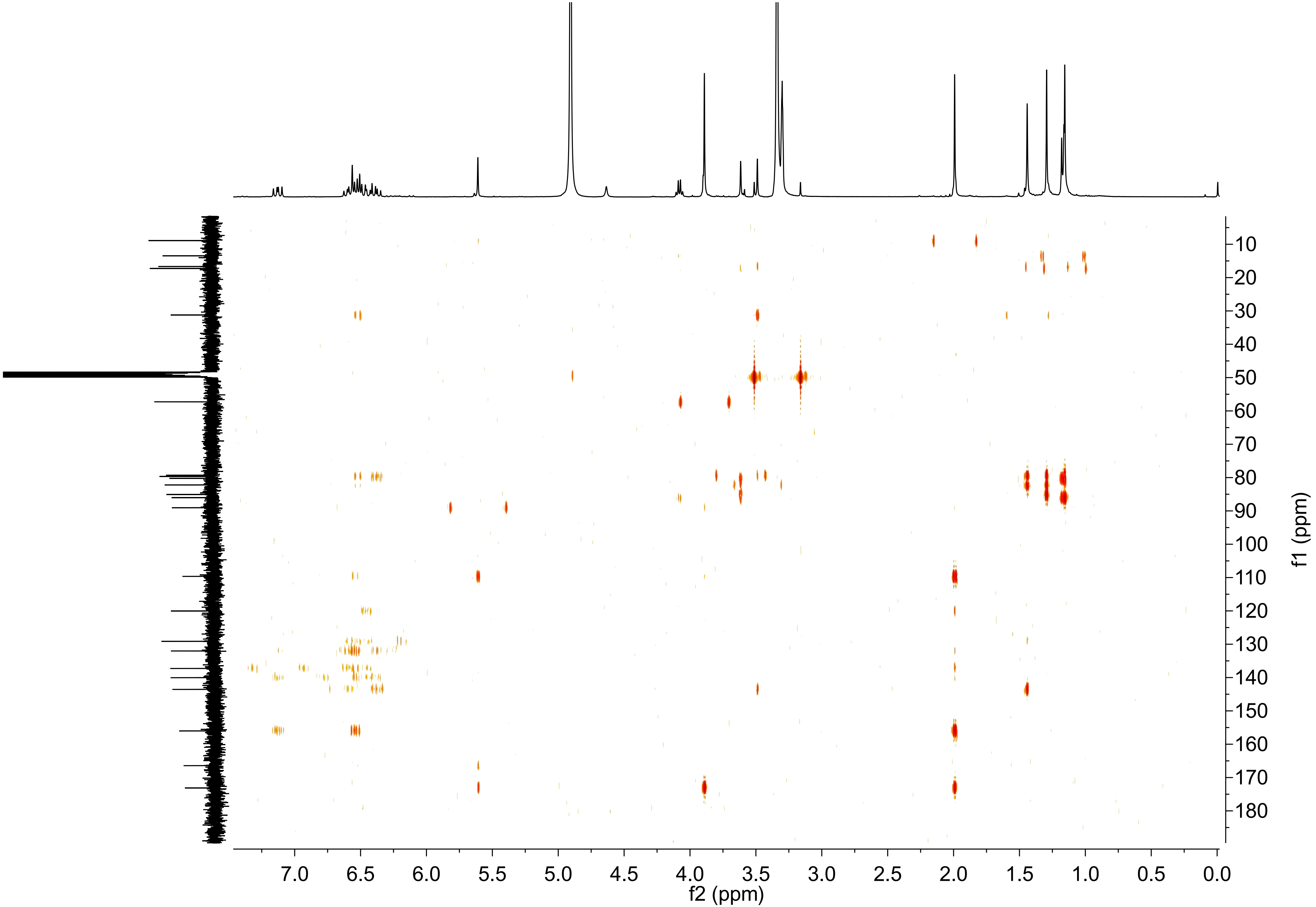

Figure S28. HMBC spectrum (400 MHz, CD_3OD) of citreoviridin M (**4**)

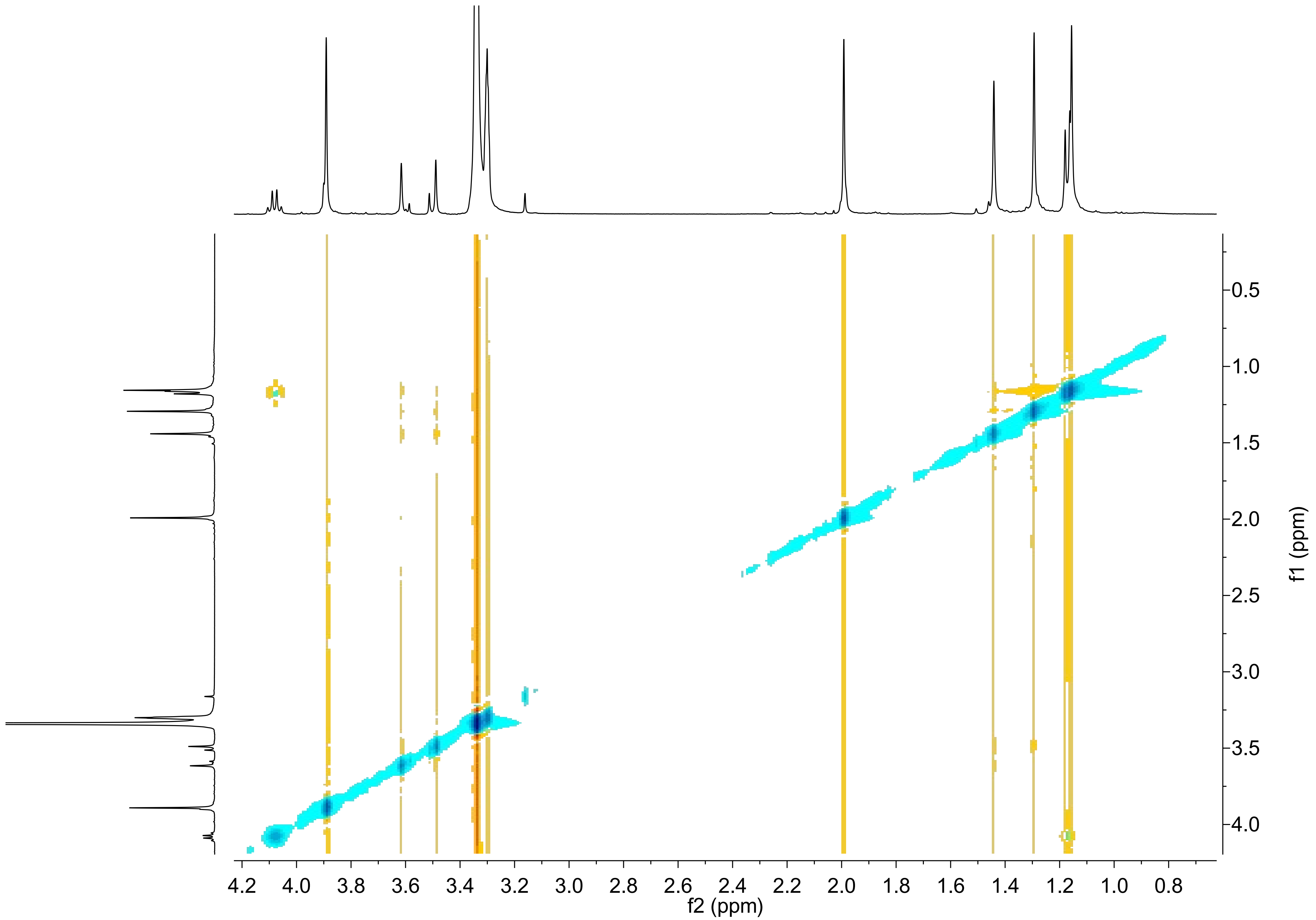

Figure S29. NOESY spectrum (400 MHz, CD_3OD) of citreoviridin M (**4**)

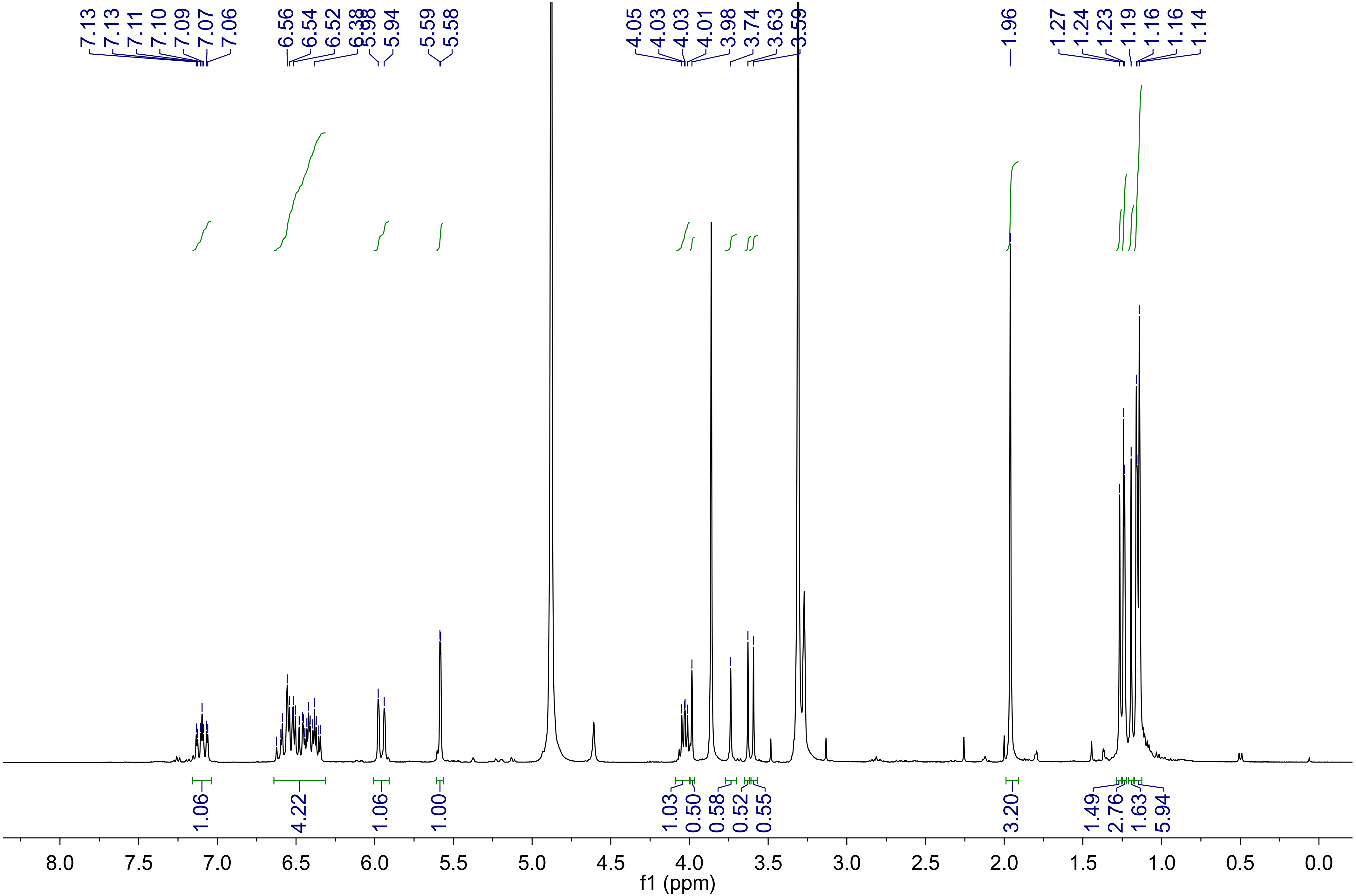

Figure S30. ^{1}H NMR spectrum (400 MHz, CD_3OD) of citreoviridins N and O (**5** and **6**)

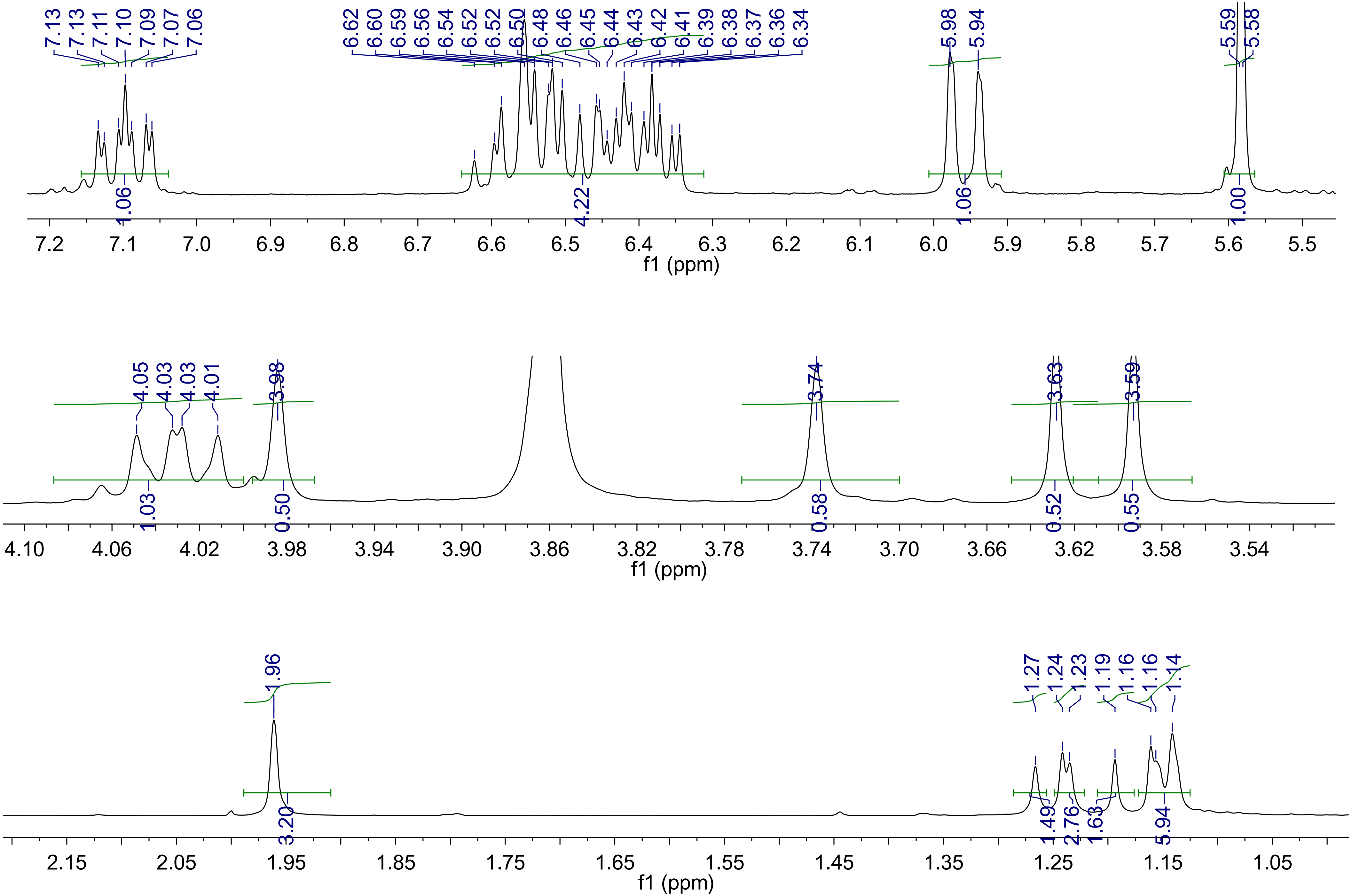

Figure S31. Amplified ^{1}H NMR spectrum (400 MHz, CD_3OD) of citreoviridins N and O (**5** and **6**)

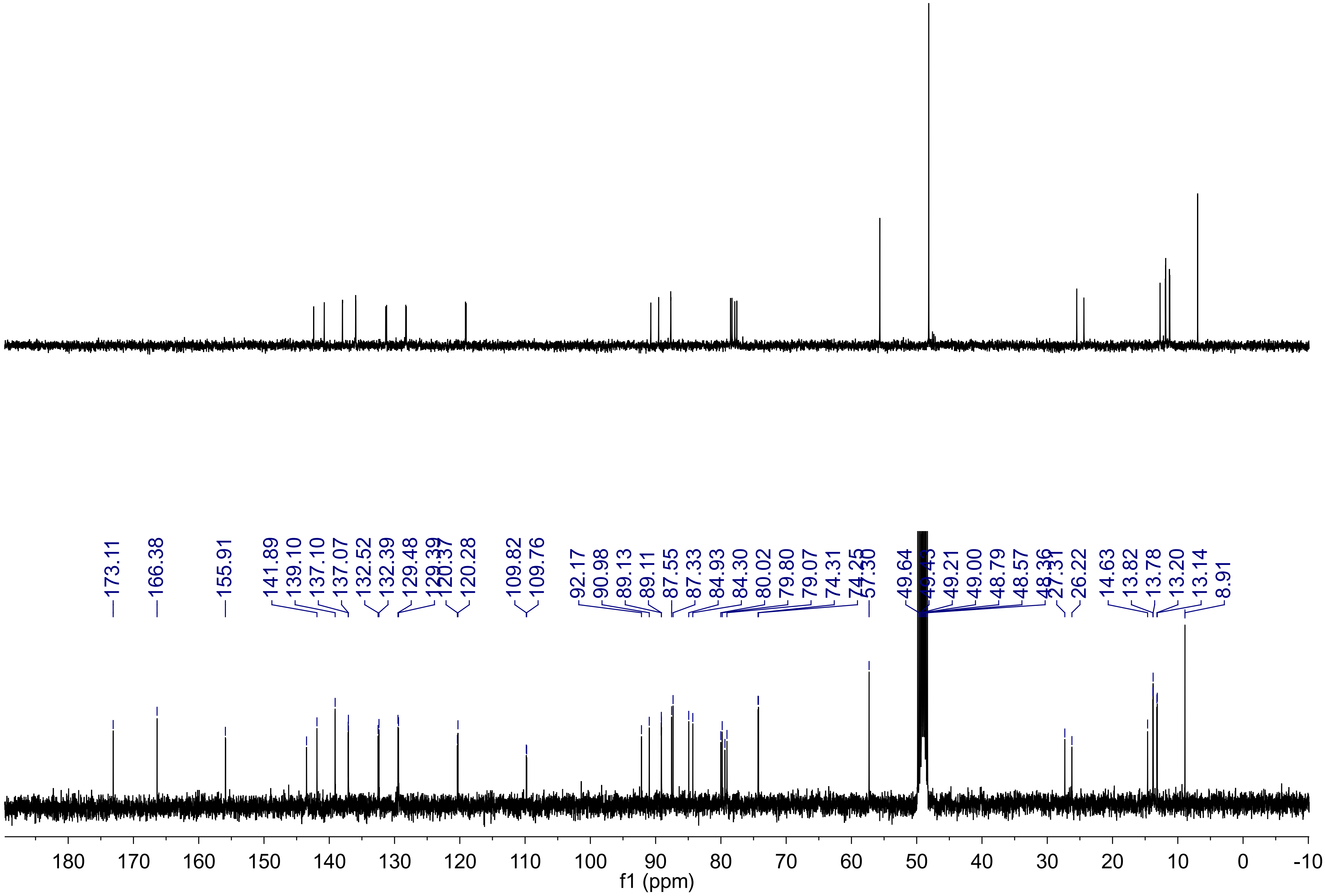

Figure S32. ^{13}C NMR and DEPT (100 MHz, CD_3OD) of citreoviridins N and O (**5** and **6**)

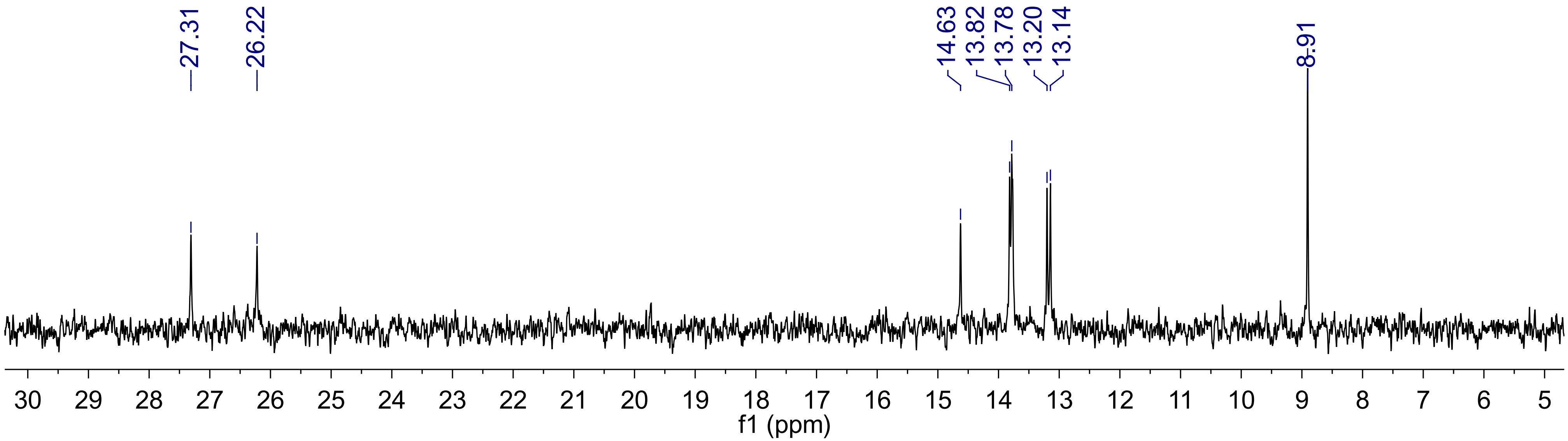

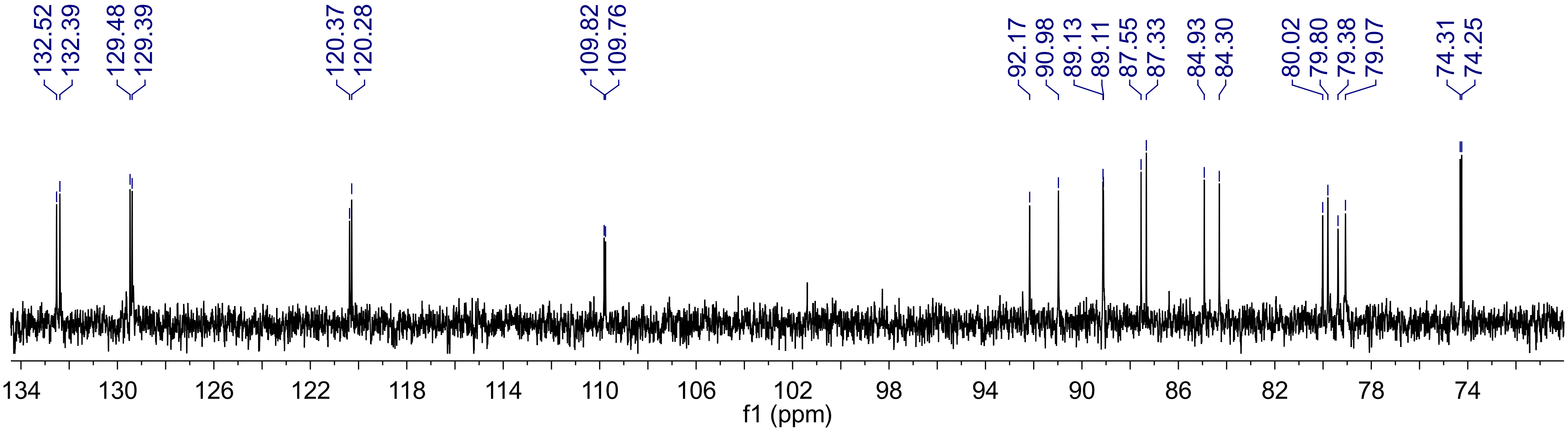

Figure S33. Amplified ^{13}C NMR spectrum (100 MHz, CD_3OD) of citreoviridins N and O (**5** and **6**)

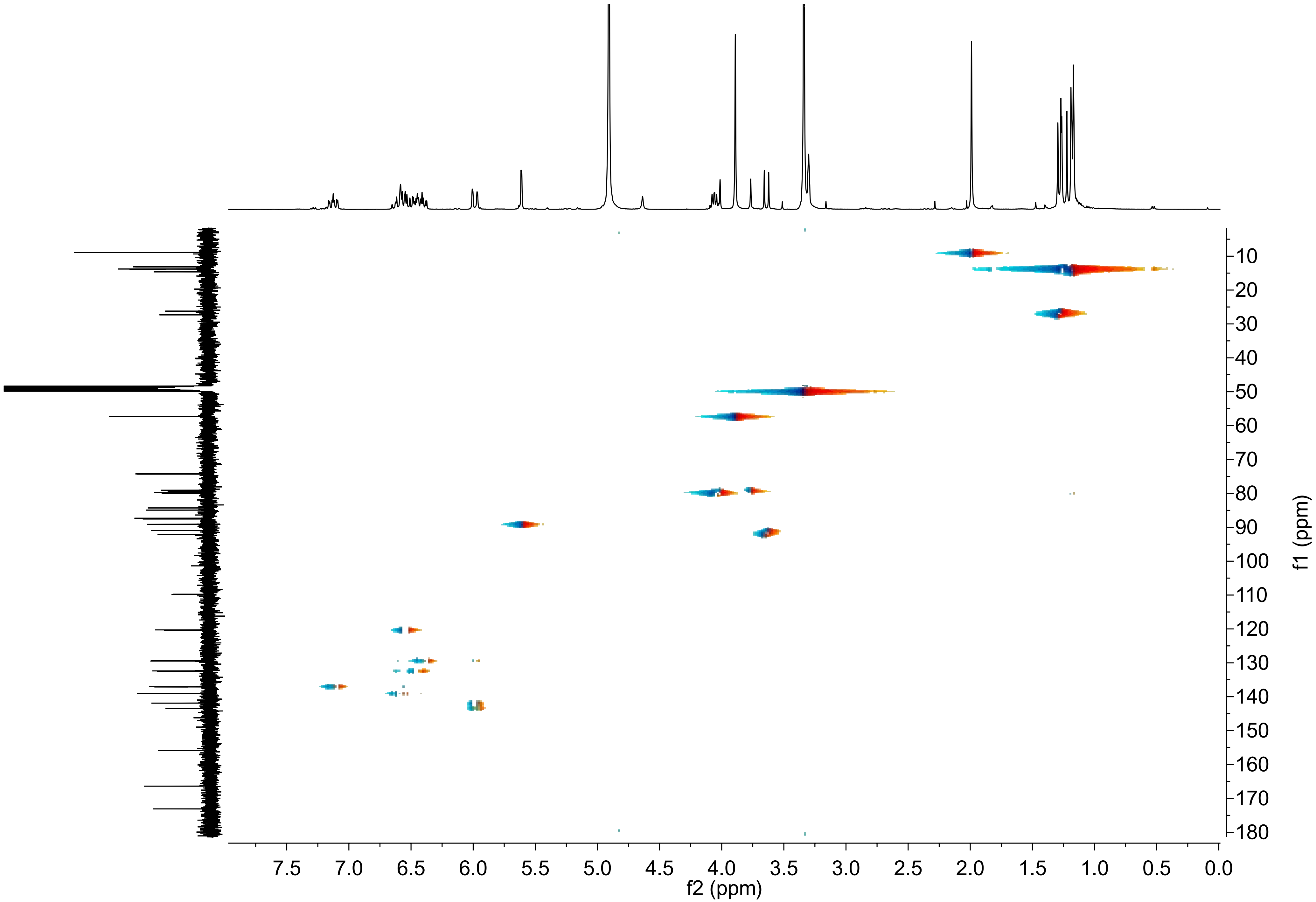

Figure S34. HMQC spectrum (400 MHz, CD_3OD) of citreoviridins N and O (**5** and **6**)

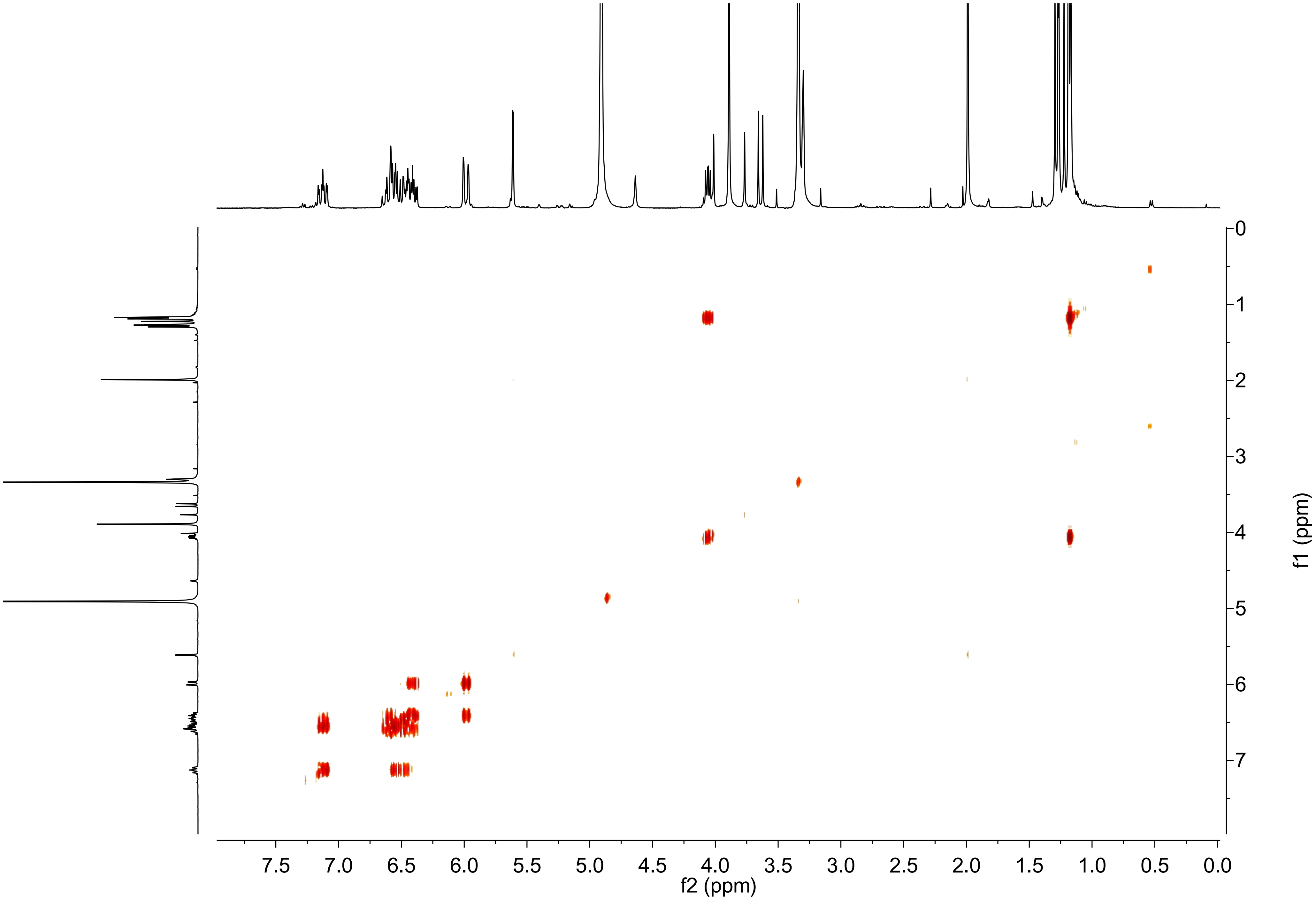

Figure S35. ^{1}H-^{1}H COSY spectrum (400 MHz, CD_3OD) of citreoviridins N and O (**5** and **6**)

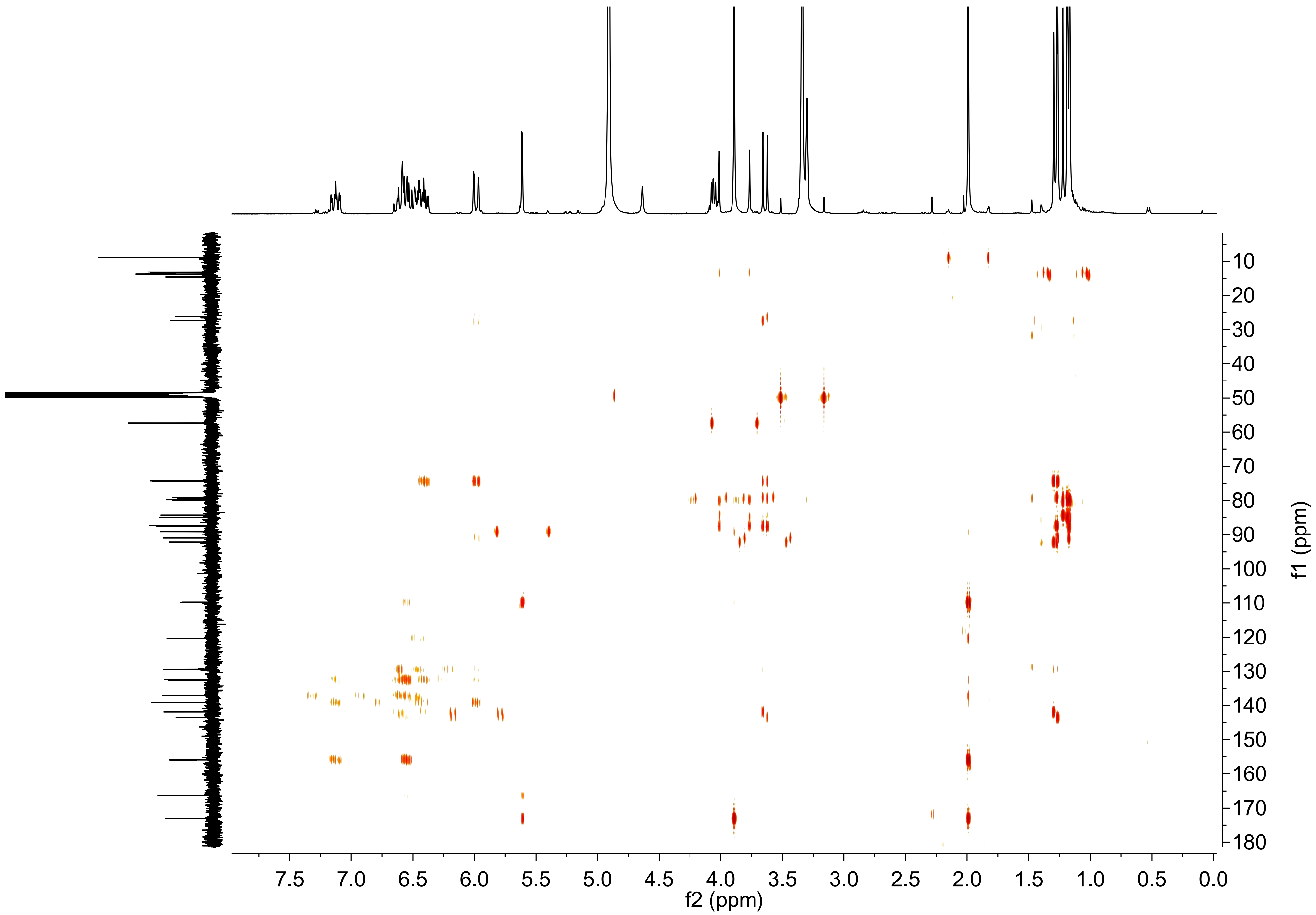

Figure S36. HMBC spectrum (400 MHz, CD_3OD) of citreoviridins N and O (**5** and **6**)

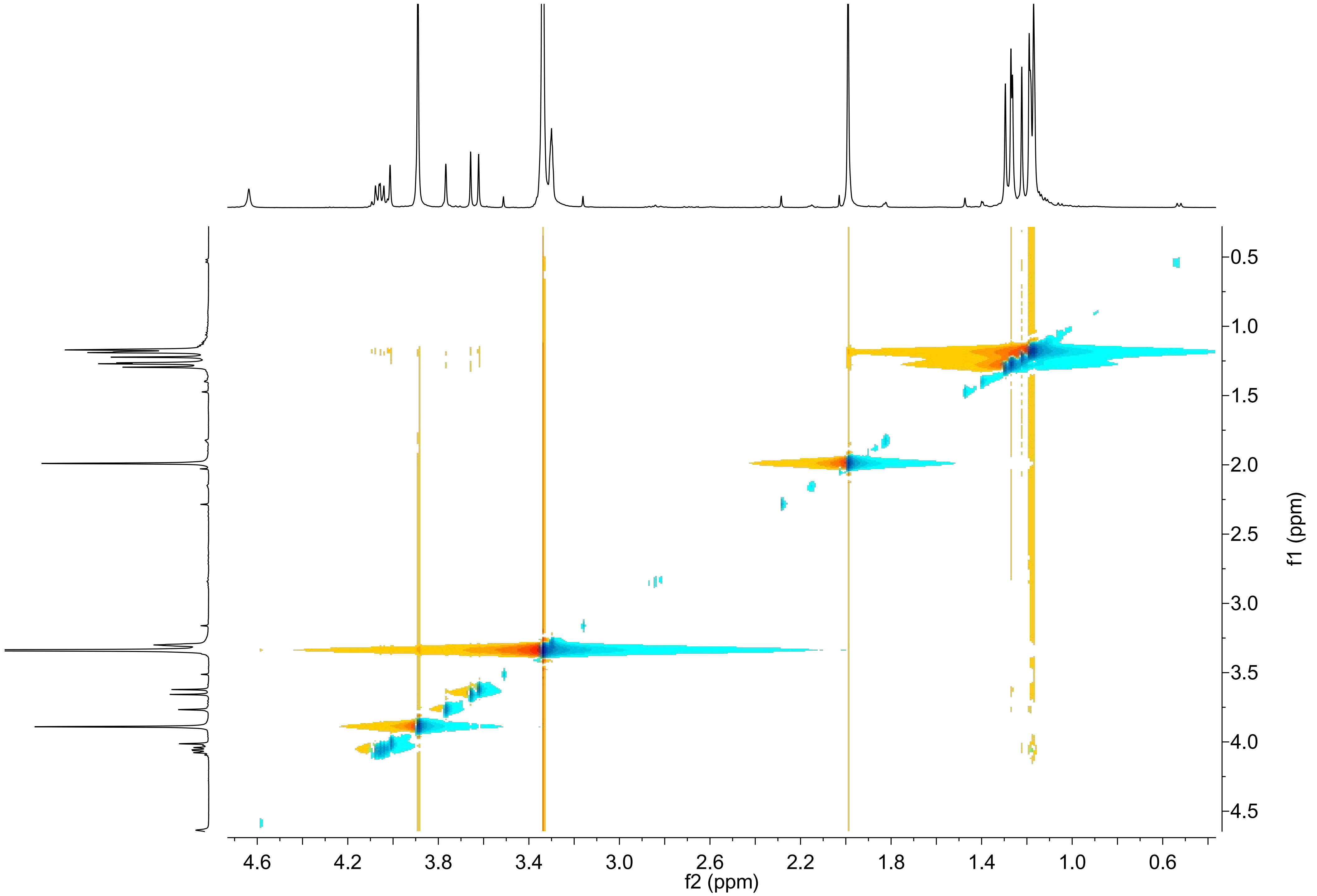

Figure S37. NOESY spectrum (400 MHz, CD_3OD) of citreoviridins N and O (**5** and **6**)

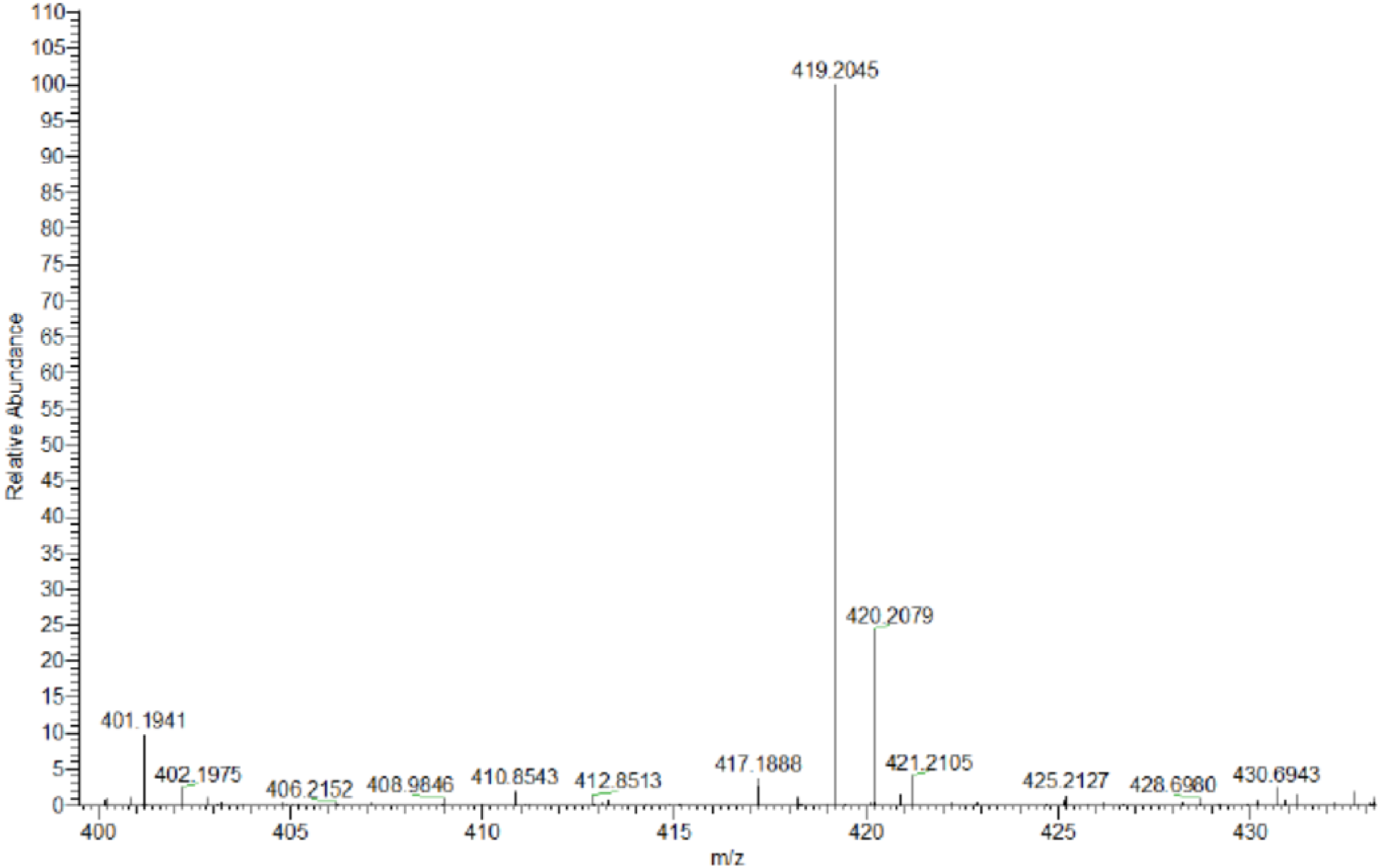

Figure S38. HR-ESI-MS of citreoviridin N (**5**)

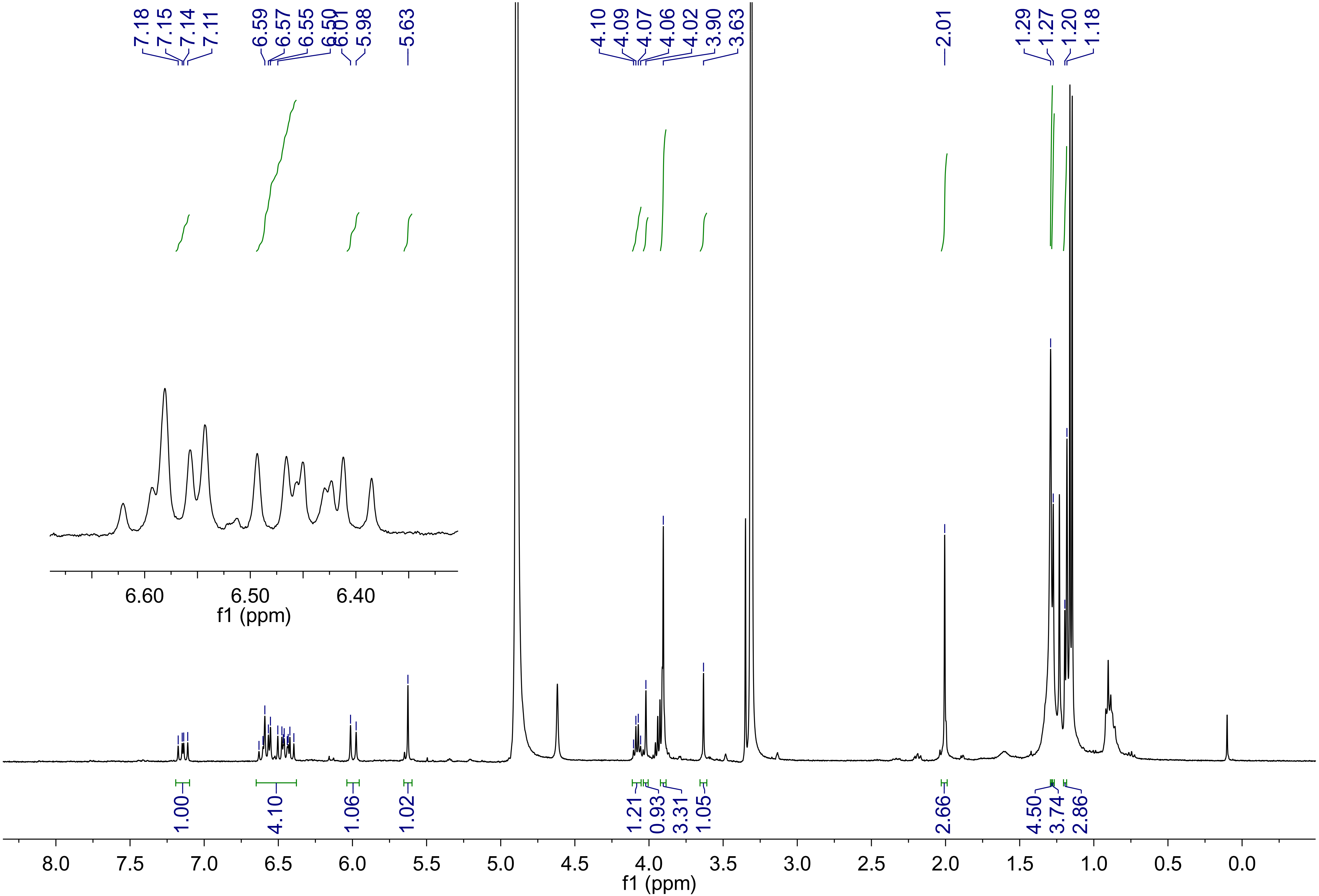

Figure S39. ^{1}H NMR spectrum (400 MHz, CD_3OD) of citreoviridin N (**5**)

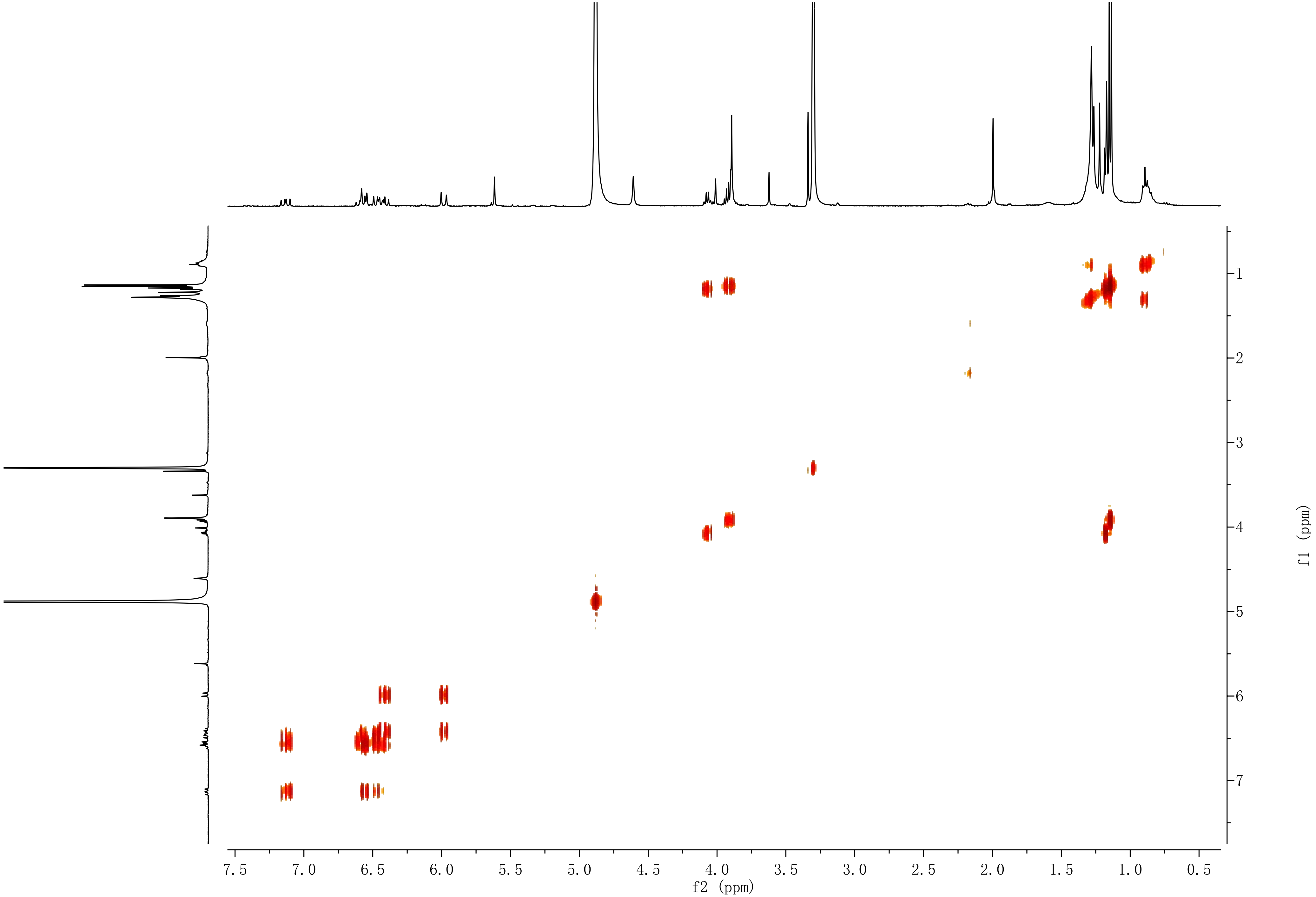

Figure S40. ^1H-^1H COSY spectrum (400 MHz, CD_3OD) of citreoviridin N (**5**)

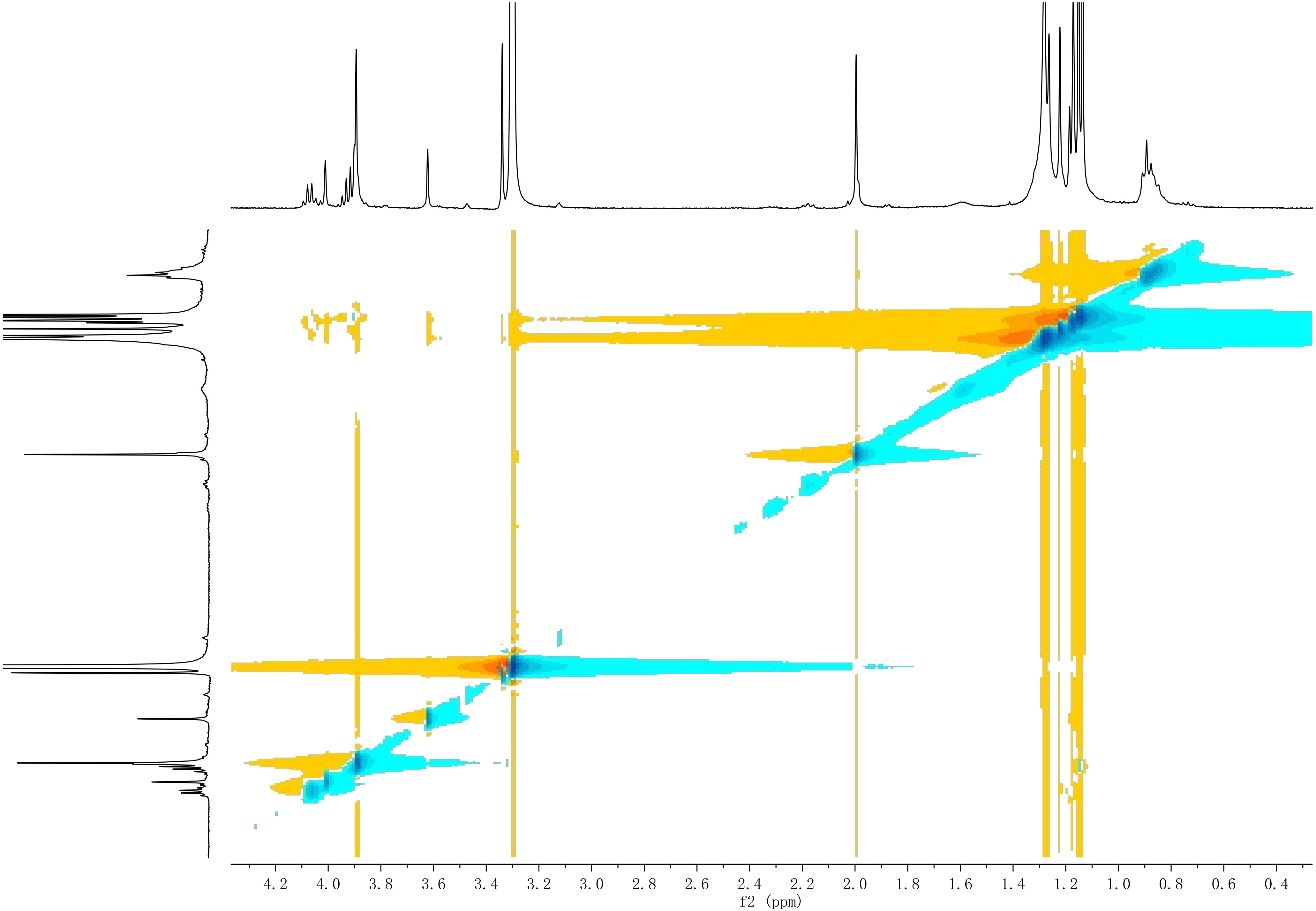

Figure S41. NOESY spectrum (400 MHz, CD_3OD) of citreoviridin N (**5**)

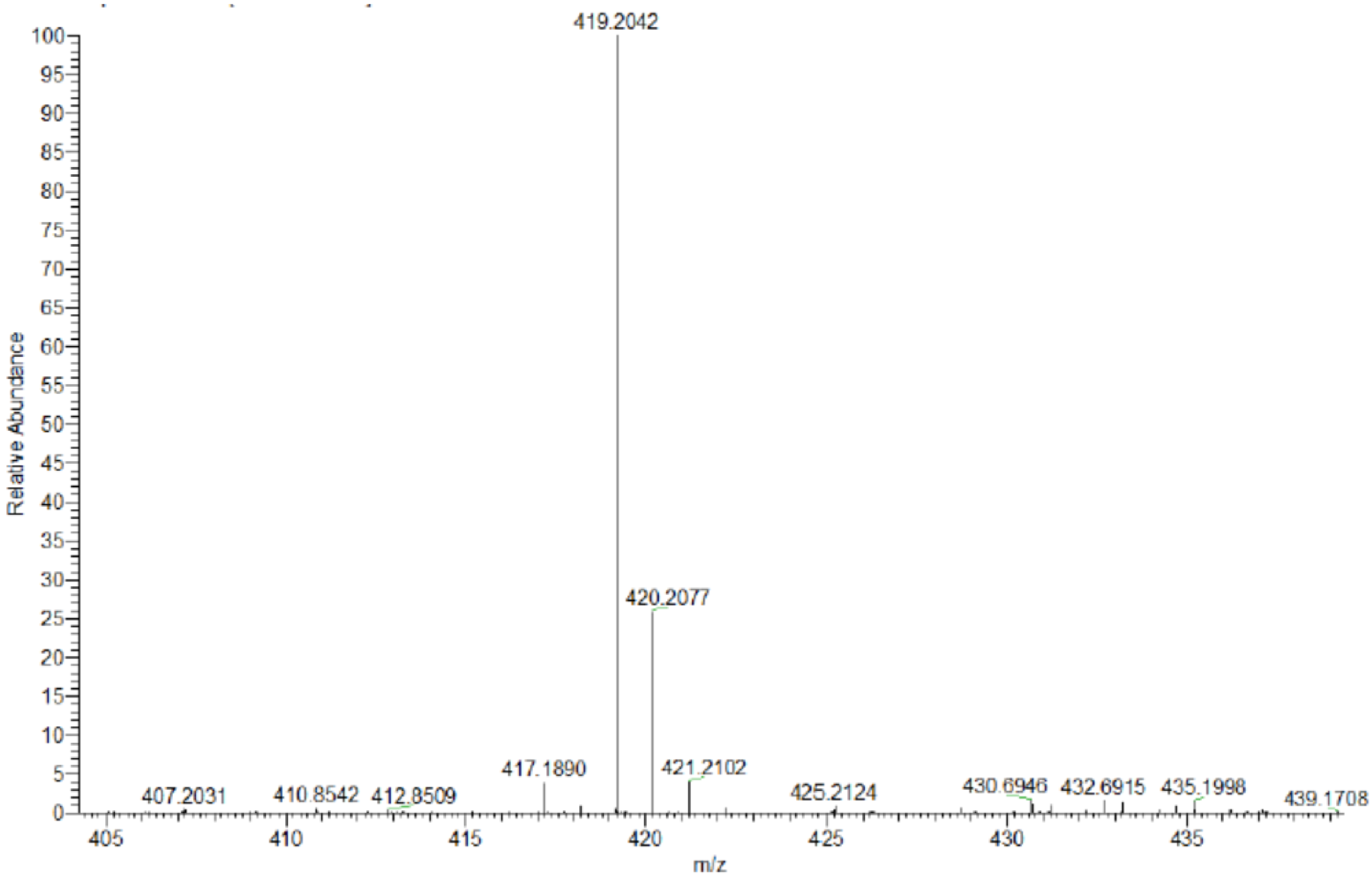

Figure S42. HR-ESI-MS of citreoviridin O (**6**)

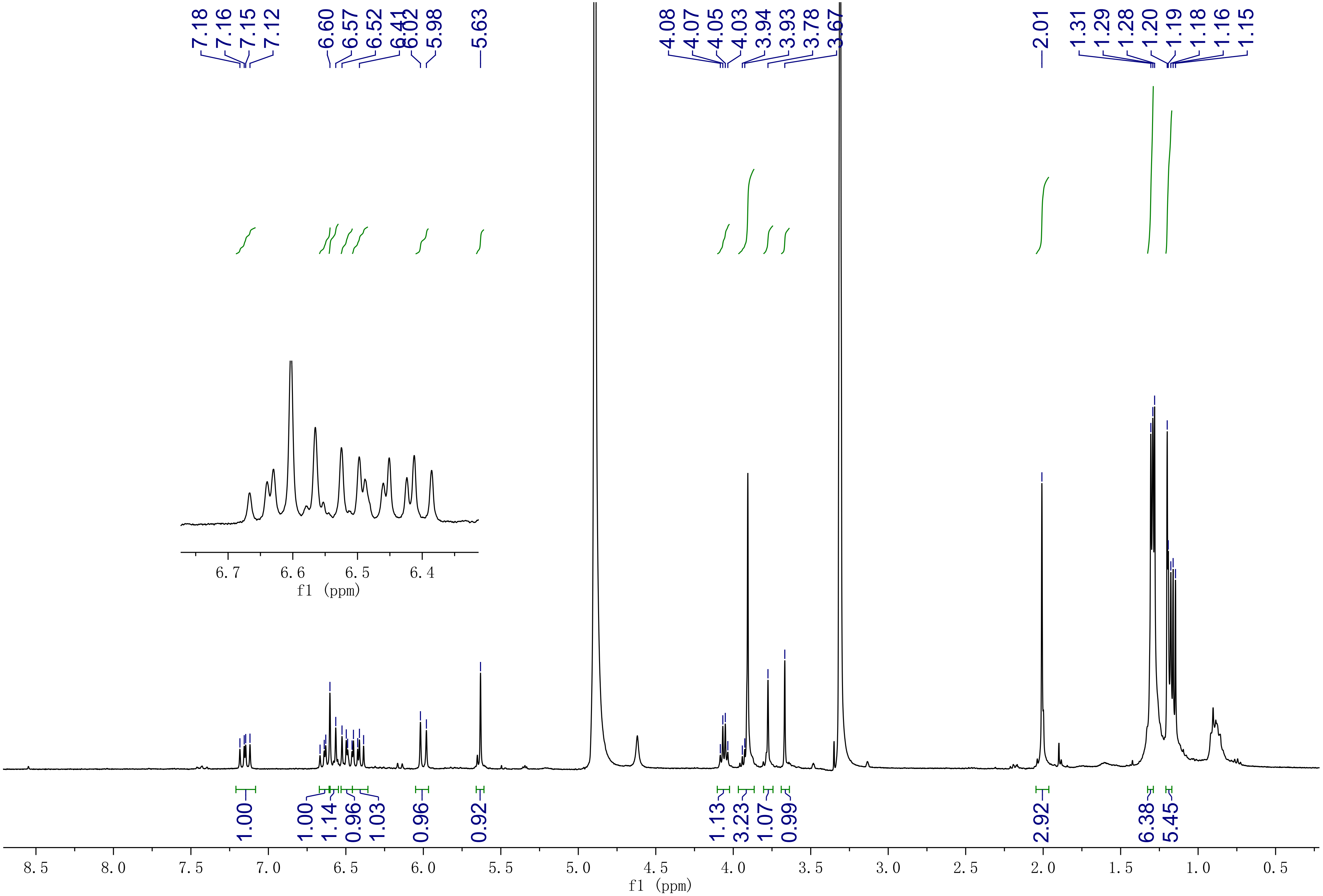

Figure S43. ^{1}H NMR spectrum (400 MHz, CD_3OD) of citreoviridin O (**6**)

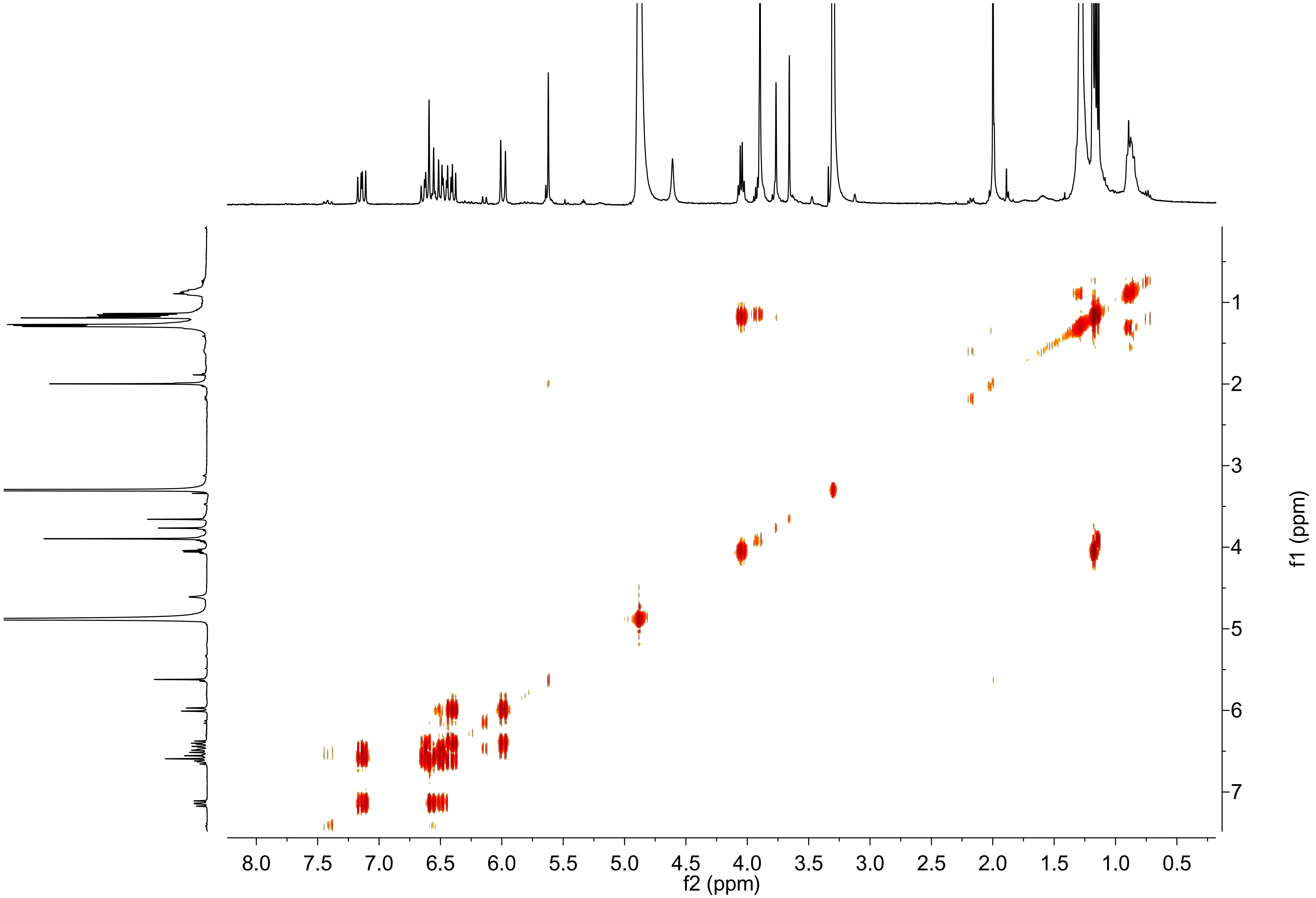

Figure S44. ^1H-^1H COSY spectrum (400 MHz, CD_3OD) of citreoviridin O (**6**)

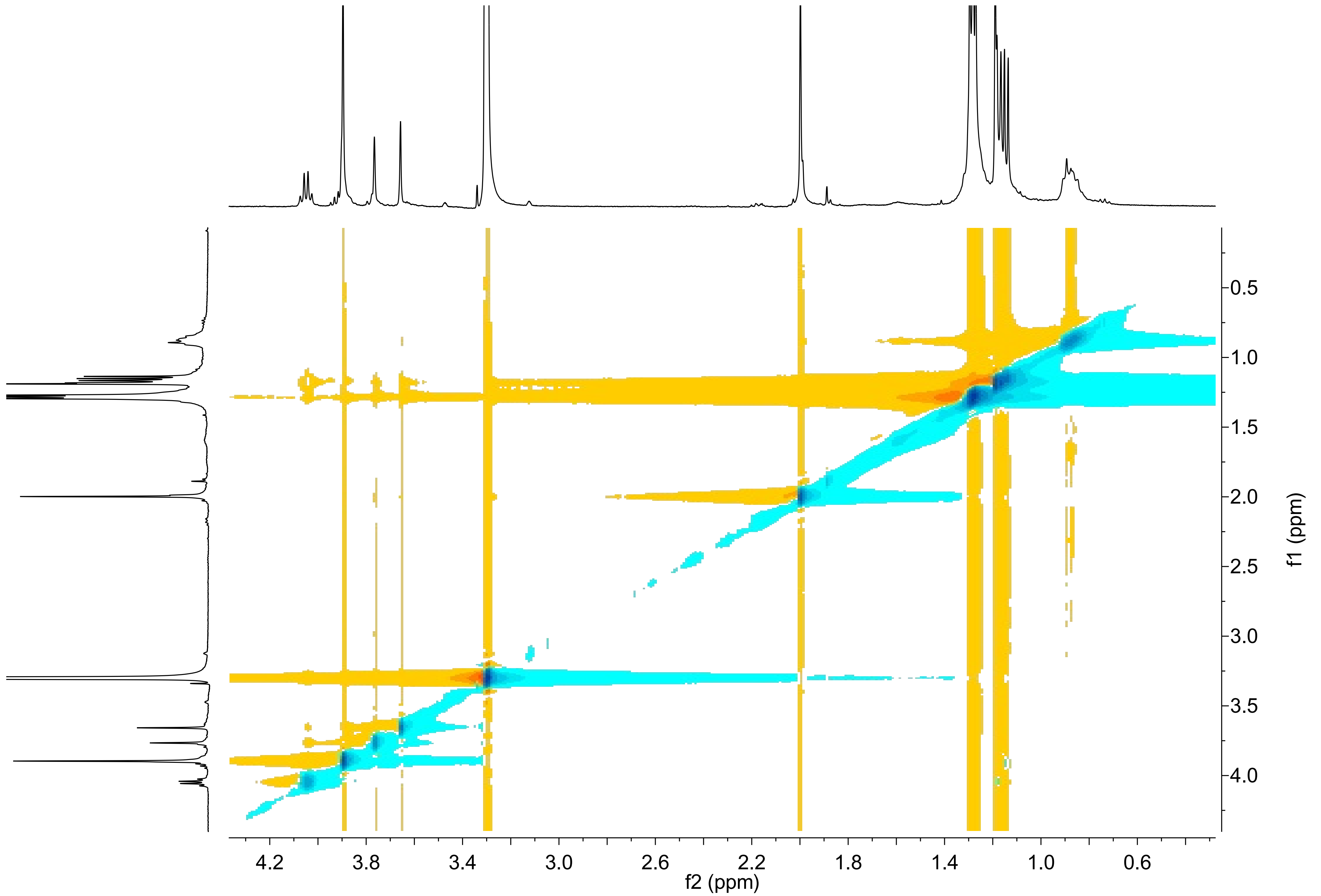

Figure S45. NOESY spectrum (400 MHz, CD_3OD) of citreoviridin O (**6**)

Single Mass Analysis

Tolerance = 10.0 mDa / DBE: min = -1.5, max = 50.0
Element prediction: Off
Number of isotope peaks used for i-FIT = 3

Monoisotopic Mass, Even Electron Ions
1 formula(e) evaluated with 0 results within limits (up to 50 closest results for each mass)
Elements Used:
C: 55-64 H: 0-200 N: 0-15 O: 0-20 Na: 0-1
ZBB-2-Dec 103 (0.408) Cm (97:104)
1: TOF MS ES+

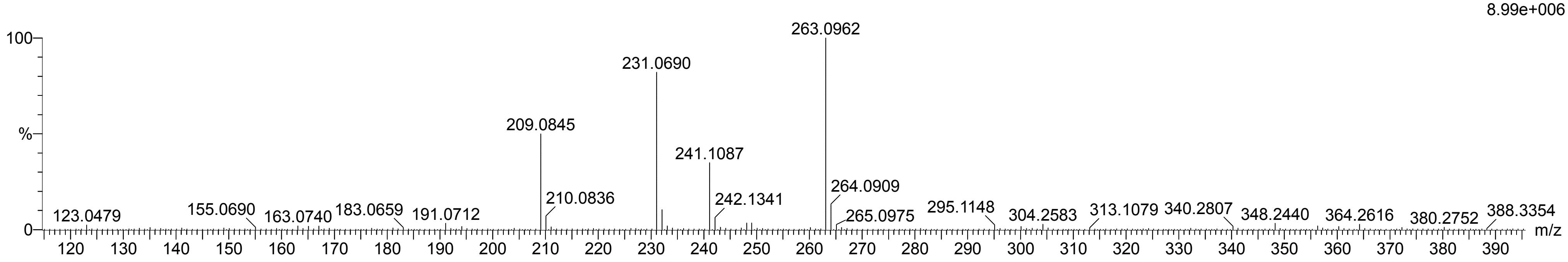


```
Minimum:                              -1.5
Maximum:               10.0    10.0    50.0

Mass        Calc. Mass  mDa     PPM     DBE     i-FIT    Norm    Conf(%)  Formula
0.0000      ---
```

Figure S46. HR-ESI-MS of pyrenocine A (**7**)

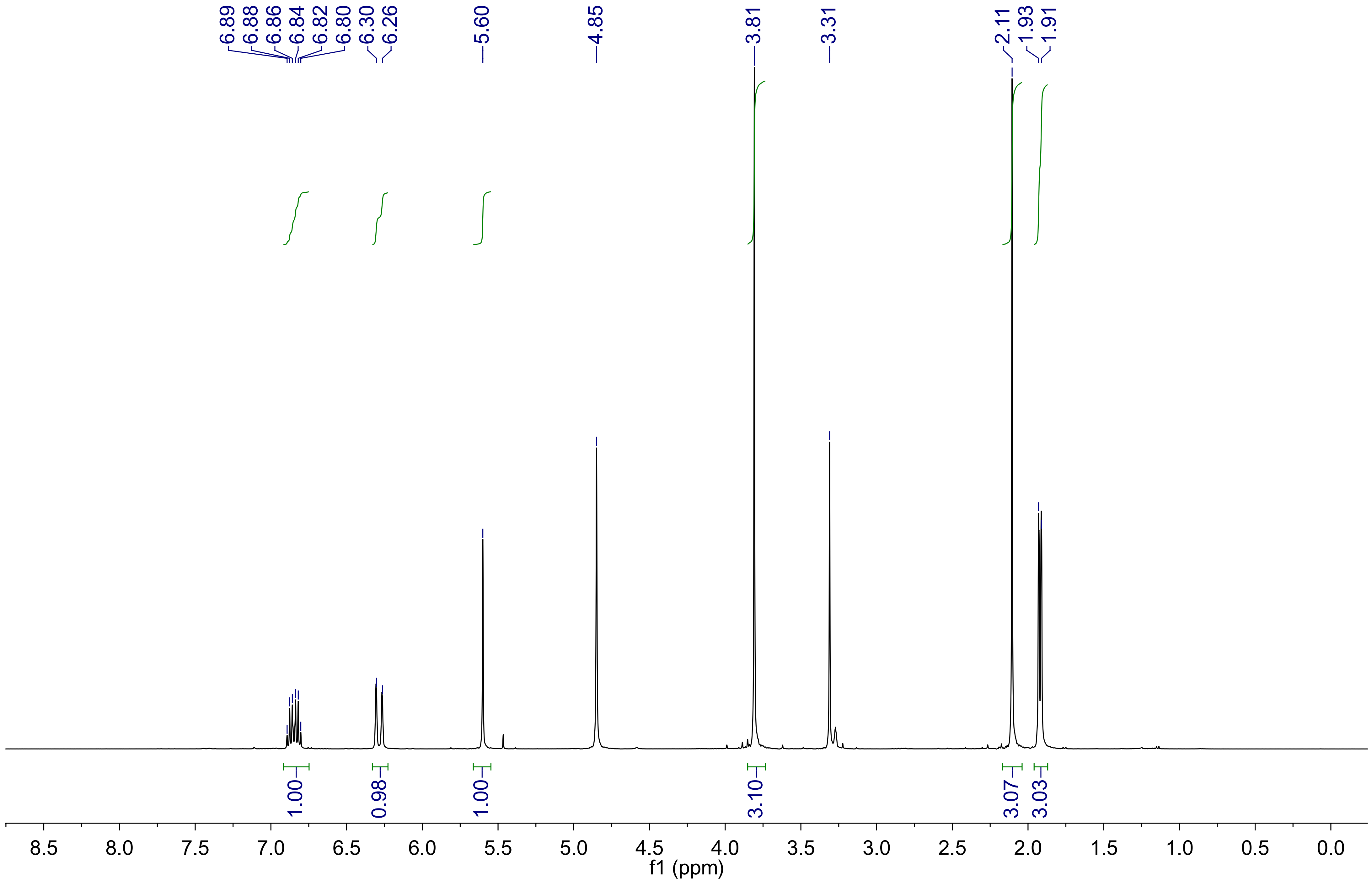

Figure S47. ^{1}H NMR spectrum (400 MHz, CD_3OD) of pyrenocine A (**7**)

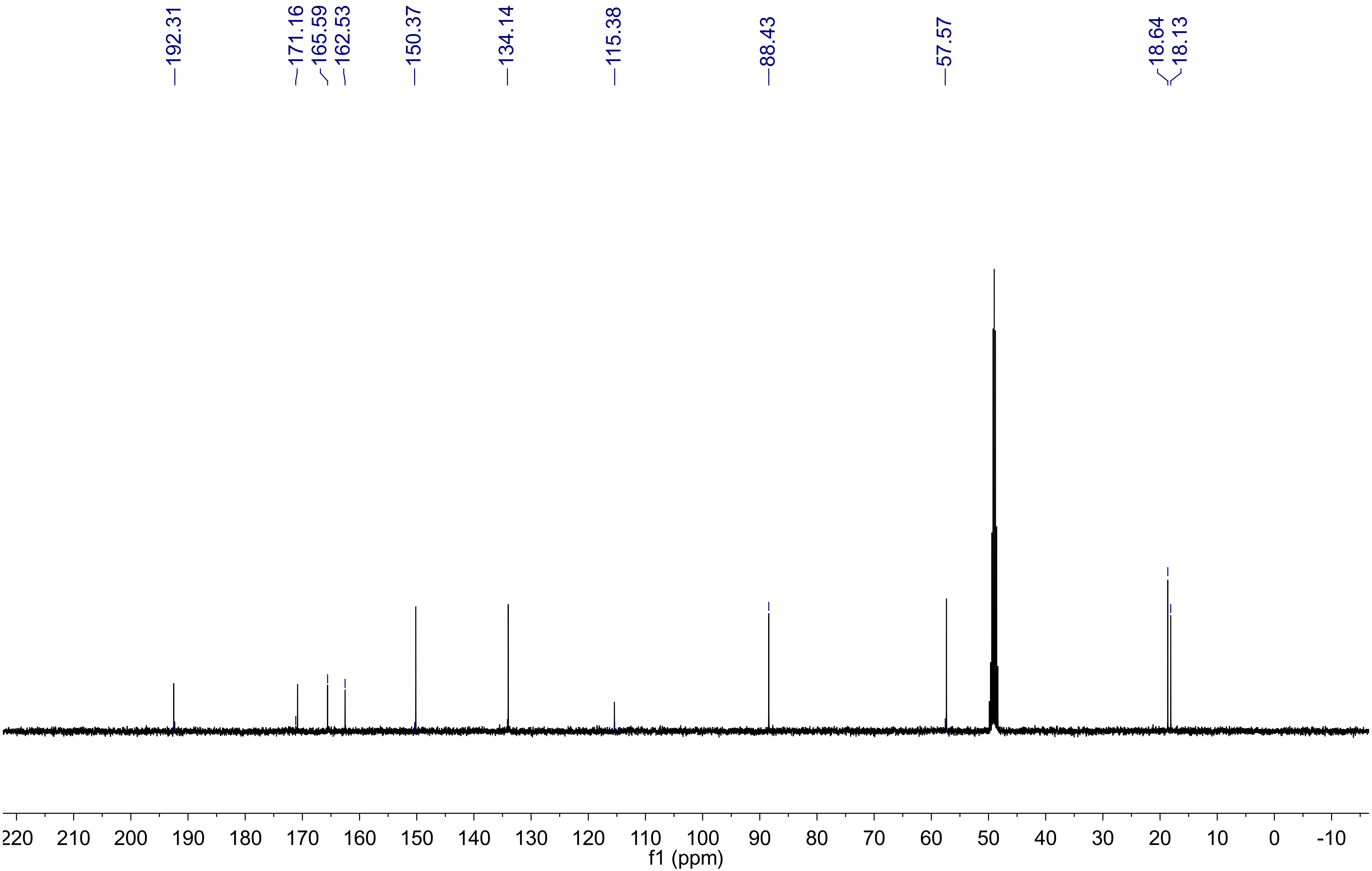

Figure S48. ^{13}C NMR spectrum (100 MHz, CD_3OD) of pyrenocine A (**7**)

Single Mass Analysis

Tolerance = 10.0 mDa / DBE: min = -1.5, max = 50.0
Element prediction: Off
Number of isotope peaks used for i-FIT = 3

Monoisotopic Mass, Even Electron Ions
1 formula(e) evaluated with 0 results within limits (up to 50 closest results for each mass)
Elements Used:
C: 10-50 H: 0-200 N: 0-15 O: 0-20 Na: 0-1
ZBB-13-Dec 110 (0.433) Cm (105:112)
1: TOF MS ES+

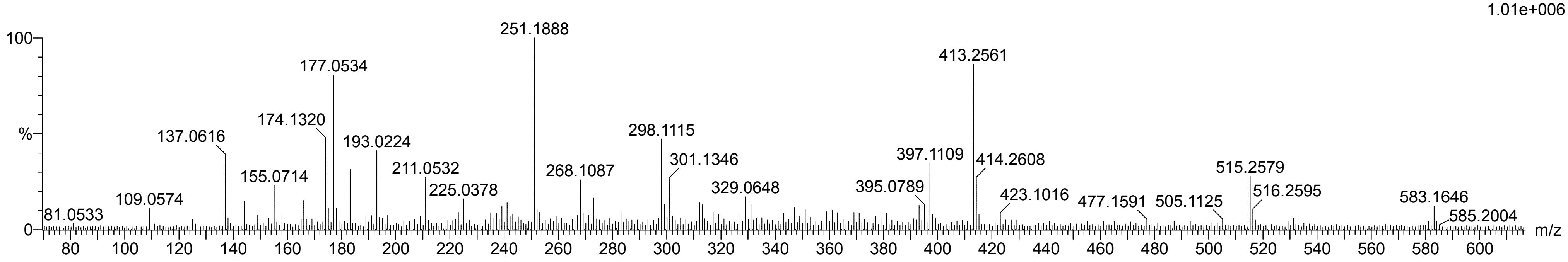

Minimum:				-1.5				
Maximum:		10.0	10.0	50.0				

Mass	Calc. Mass	mDa	PPM	DBE	i-FIT	Norm	Conf(%)	Formula
0.0000	---							

Figure S49. HR-ESI-MS of terrein (**14**)

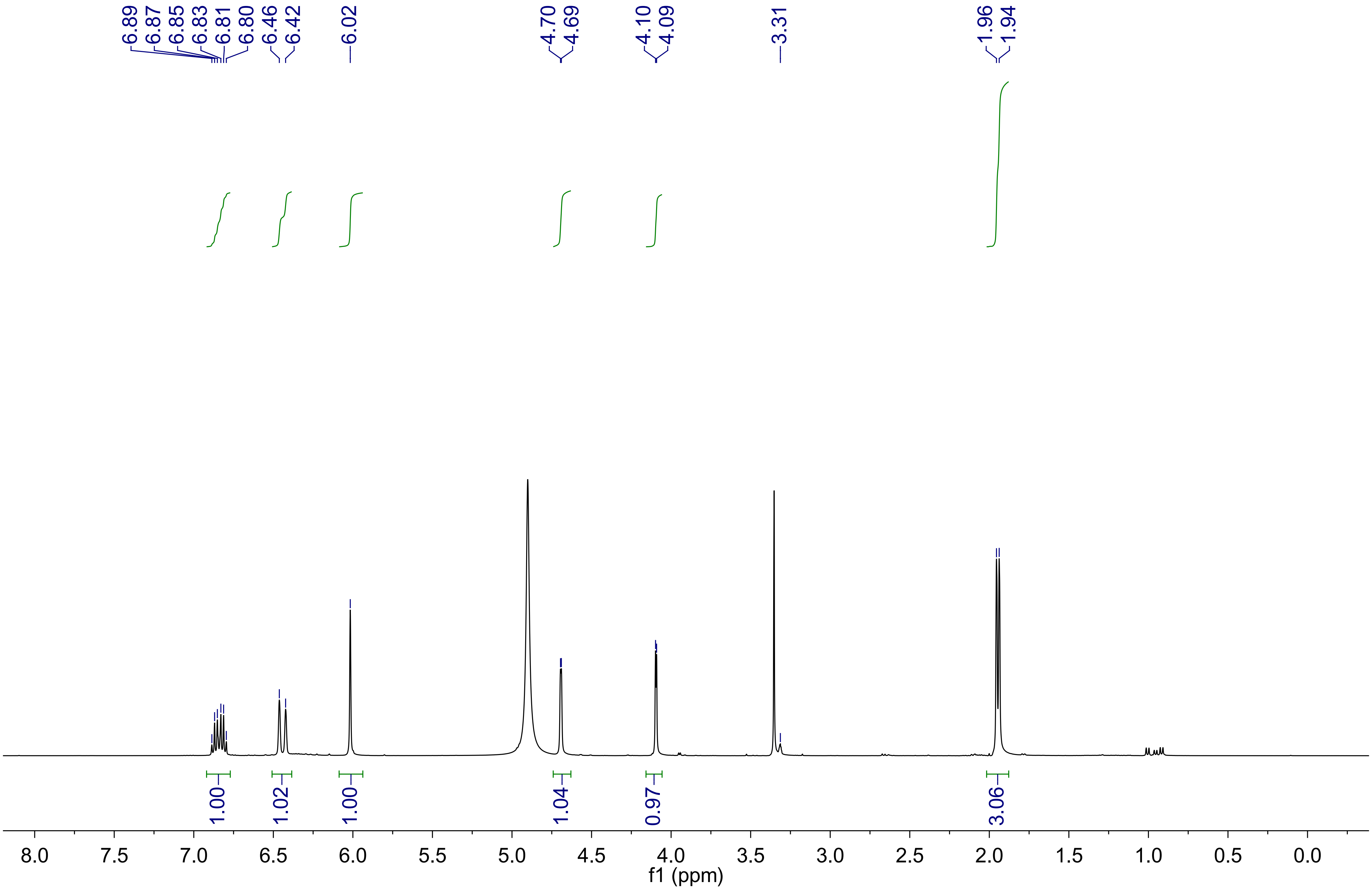

Figure S50. ^{1}H NMR spectrum (400 MHz, CD_3OD) of terrein (**14**)

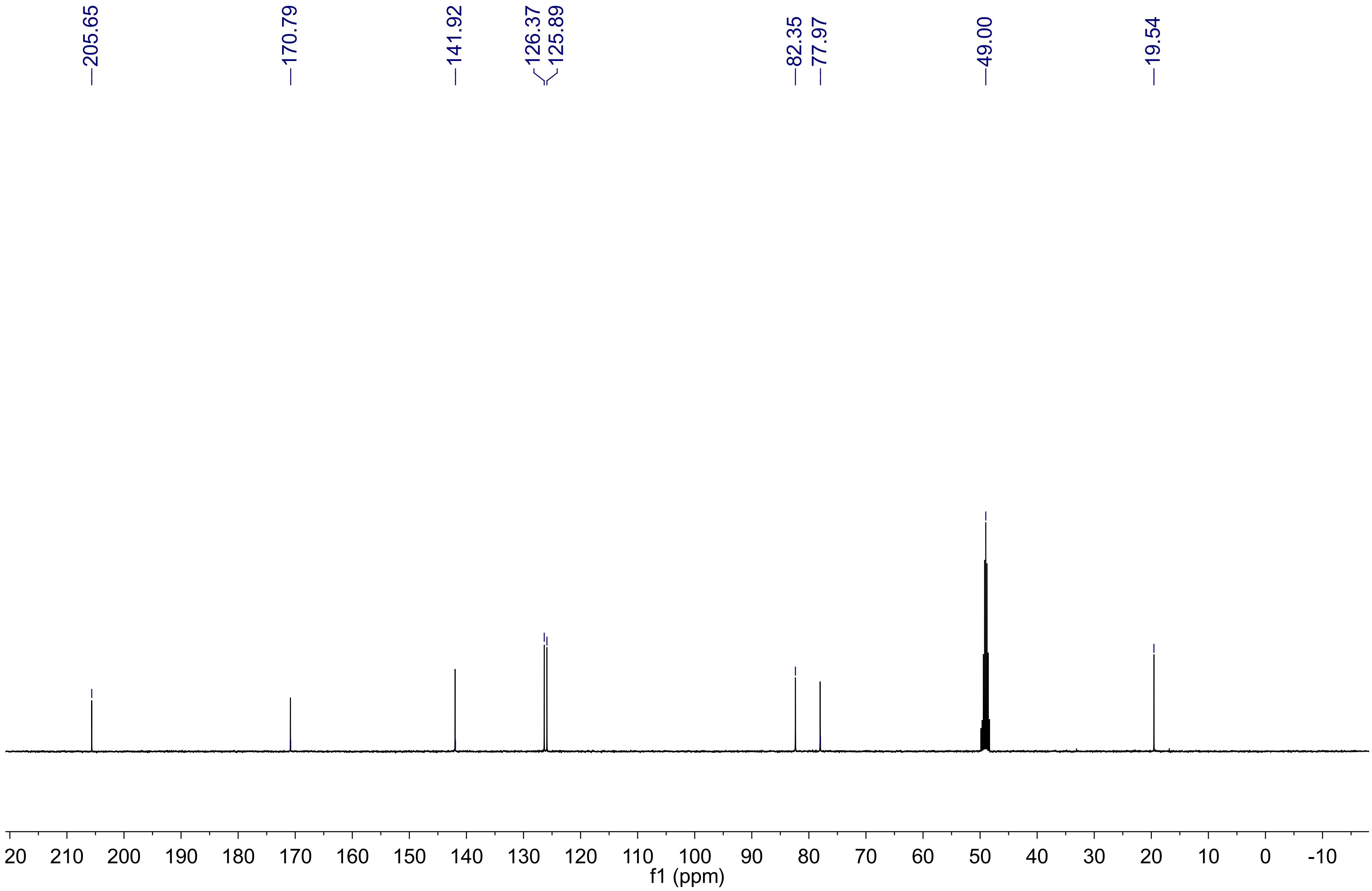

Figure S51. ^{13}C NMR spectrum (100 MHz, CD_3OD) of terrein (**14**)

Single Mass Analysis

Tolerance = 10.0 mDa / DBE: min = -10.0, max = 100.0
Element prediction: Off
Number of isotope peaks used for i-FIT = 3

Monoisotopic Mass, Even Electron Ions
1 formula(e) evaluated with 0 results within limits (all results (up to 1000) for each mass)
Elements Used:

ZBB-23-Jan 101 (0.400) Cm (101:102)
1: TOF MS ES+

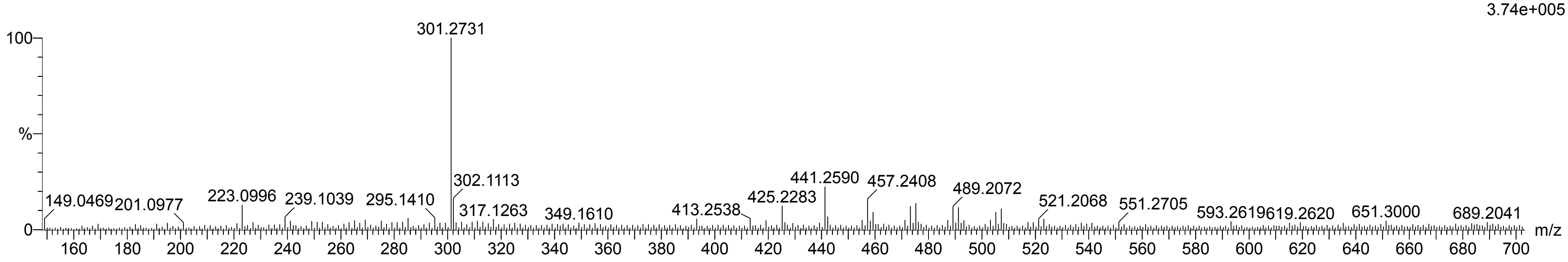

		mDa	PPM	DBE	i-FIT	Norm	Conf(%)	Formula
Minimum:				-10.0				
Maximum:		10.0	10.0	100.0				

Mass	Calc. Mass	mDa	PPM	DBE	i-FIT	Norm	Conf(%)	Formula
0.0000	---							

Figure S52. HR-ESI-MS of citreoviridin (**20**)

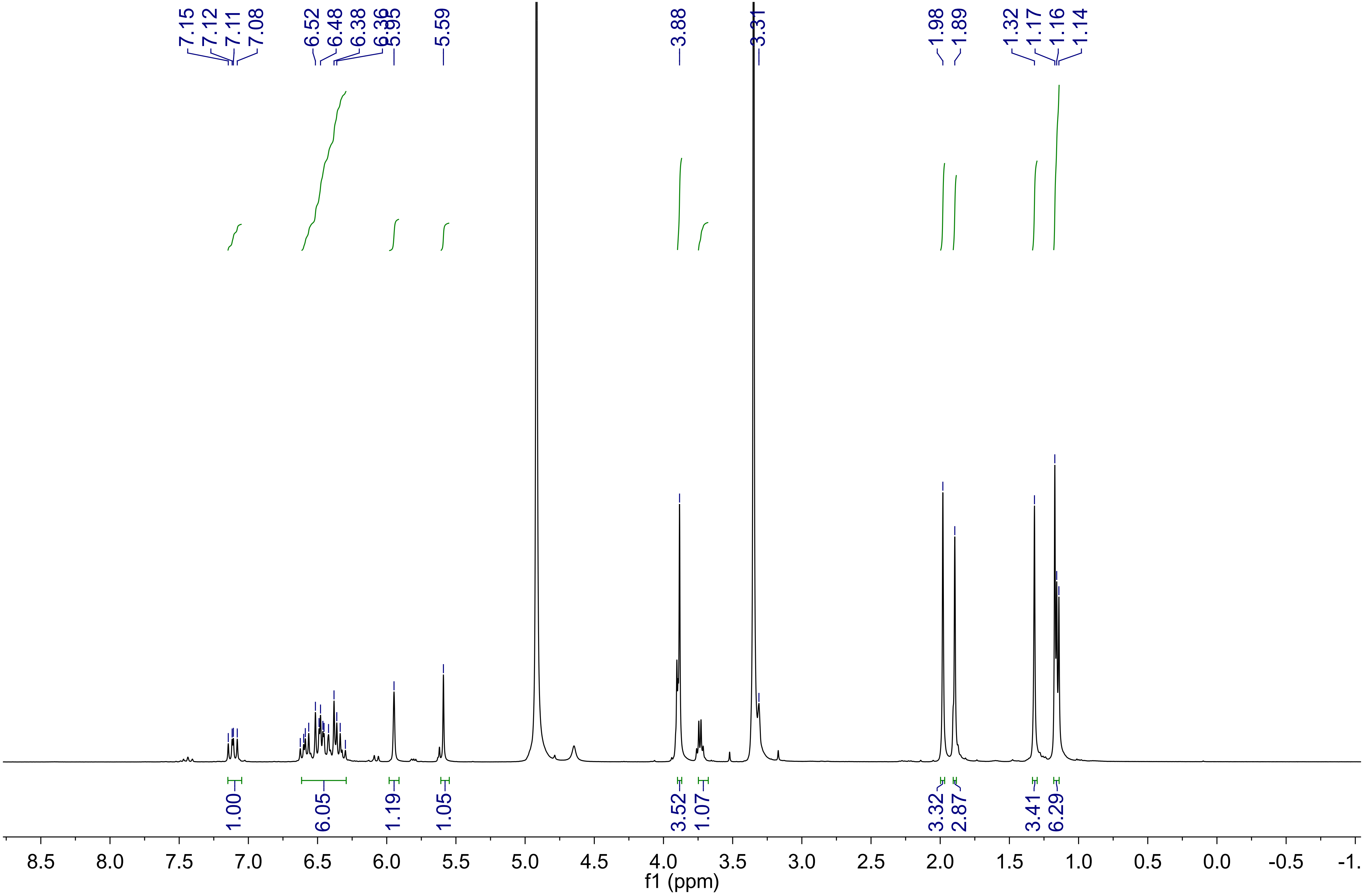

Figure S53. ^{1}H NMR spectrum (400 MHz, CD_3OD) of citreoviridin (**20**)

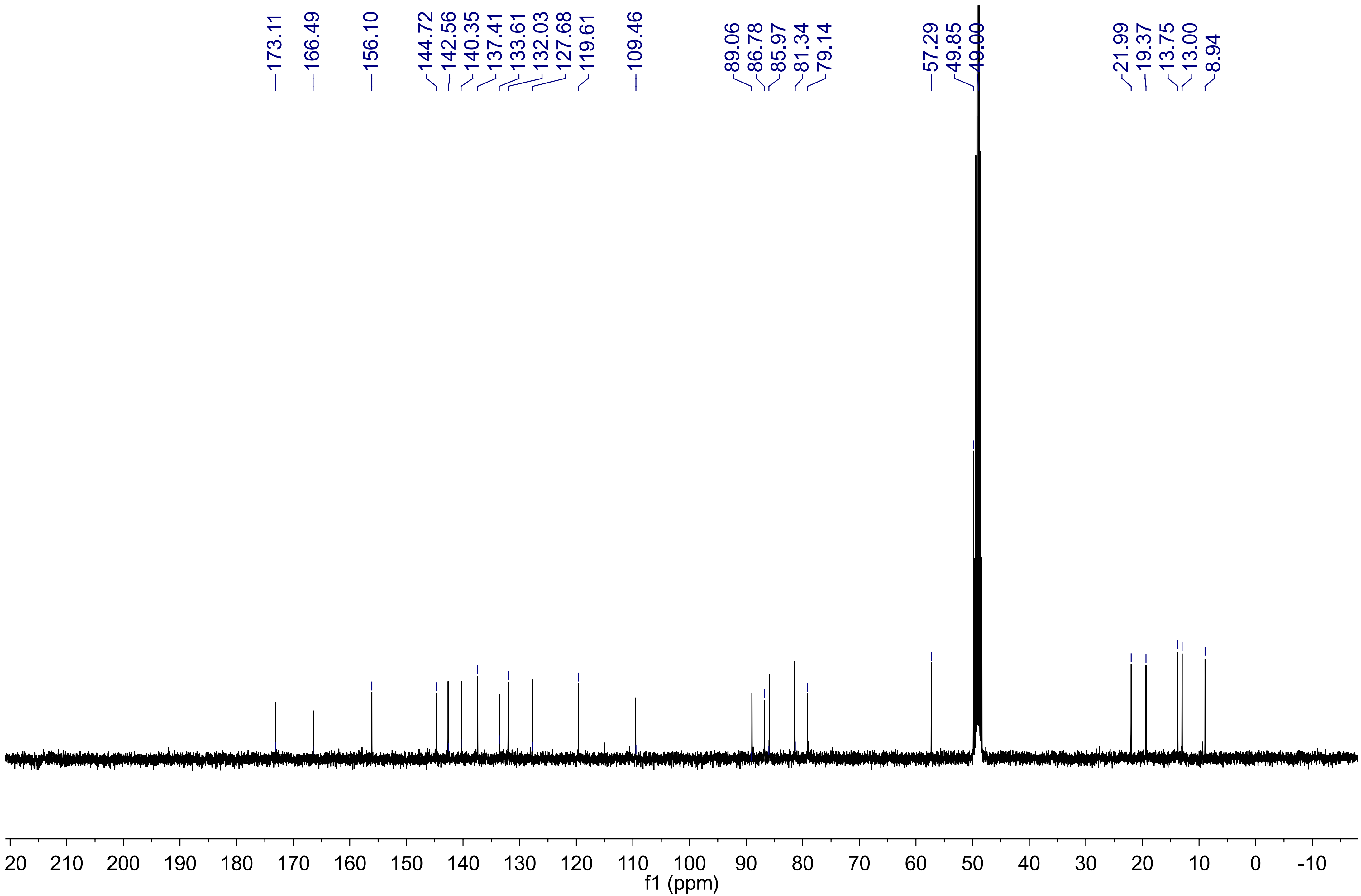

Figure S54. ^{13}C NMR spectrum (100 MHz, CD_3OD) of citreoviridin (**20**)
